# 7β-Hydroxysteroid dehydratase Hsh3 eliminates the 7-hydroxy group of the bile salt ursodeoxycholate during degradation by *Sphingobium* sp. strain Chol11 and other *Sphingomonadaceae*

**DOI:** 10.1128/aem.00185-25

**Published:** 2025-05-09

**Authors:** Phil Richtsmeier, Ruslan Nedielkov, Malte Haring, Onur Yücel, Lea Elsner, Rebekka Herdis Lülf, Lars Wöhlbrand, Ralf Rabus, Heiko Moeller, Bodo Philipp, Franziska Maria Mueller

**Affiliations:** 1 Microbial Biotechnology and Ecology, Institute for Molecular Microbiology and Biotechnology, University of Münster 54324, Münster, Germany; 2 Institute for Chemistry, University of Potsdam 426790, Potsdam, Germany; 3 General and Molecular Microbiology, Institute for Chemistry and Biology of the Marine Environment (ICBM), Carl von Ossietzky Universität Oldenburg 436553, Oldenburg, Germany; 4 Applied Ecology and Bioresources, Fraunhofer-Institute for Molecular and Applied Ecology IME 683029 , Schmallenberg, Germany; Danmarks Tekniske Universitet The Novo Nordisk Foundation Center for Biosustainability, Kgs. Lyngby, Denmark

**Keywords:** ursodeoxycholate, steroid degradation, steroid transformation, hydroxysteroid dehydratase

## Abstract

Bile salts are steroids with distinctive hydroxylation patterns that are produced and excreted by vertebrates. In contrast to common human bile salts, ursodeoxycholate (UDCA) has a 7-hydroxy group in β-configuration and is used in large amounts for the treatment of diverse gastrointestinal diseases. We isolated the 7β-hydroxysteroid dehydratase Hsh3 that is involved in UDCA degradation by *Sphingobium* sp. strain Chol11. Hsh3 eliminates the 7β-hydroxy group as water, leading to a double bond in the B-ring. This is similar to 7α-hydroxysteroid dehydratases in this and other strains, but Hsh3 is evolutionarily different from these. Purified Hsh3 accepted steroids with and without side chains as substrates and had minor activity with 7α-hydroxy groups. The deletion mutant strain Chol11 Δ*hsh3* had impacted growth with UDCA and accumulated a novel compound. The compound was identified as 3′,5-dihydroxy-H-methyl-hexahydro-5-indene-1-one-propanoate, consisting of steroid rings C and D with a C_3_-side chain carrying the former 7β-hydroxy group, indicating that Hsh3 activity is important especially for the later stages of bile salt degradation. Hsh3 homologs were found in other sphingomonads that were also able to degrade UDCA as well as in environmental metagenomes. Thus, Hsh3 adds to the bacterial enzyme repertoire for degrading a variety of differently hydroxylated bile salts.

**IMPORTANCE:**

The bacterial degradation of different bile salts is not only important for the removal of these steroidal compounds from the environment but also harbors interesting enzymes for steroid biotechnology. The 7β-hydroxy bile salt ursodeoxycholate (UDCA) naturally occurs in high concentration in the feces of black bears and has important pharmaceutical relevance for the treatment of different liver-related diseases, for which it is administered in high and increasing amounts. Additionally, it is present in the bile salt pool of humans in trace amounts. While UDCA degradation is environmentally important, the enzyme Hsh3 modifies the hydroxy group that confers the medically relevant properties and thus might be interesting for microbiome analyses and biotechnological applications.

## INTRODUCTION

**Fig 1:**
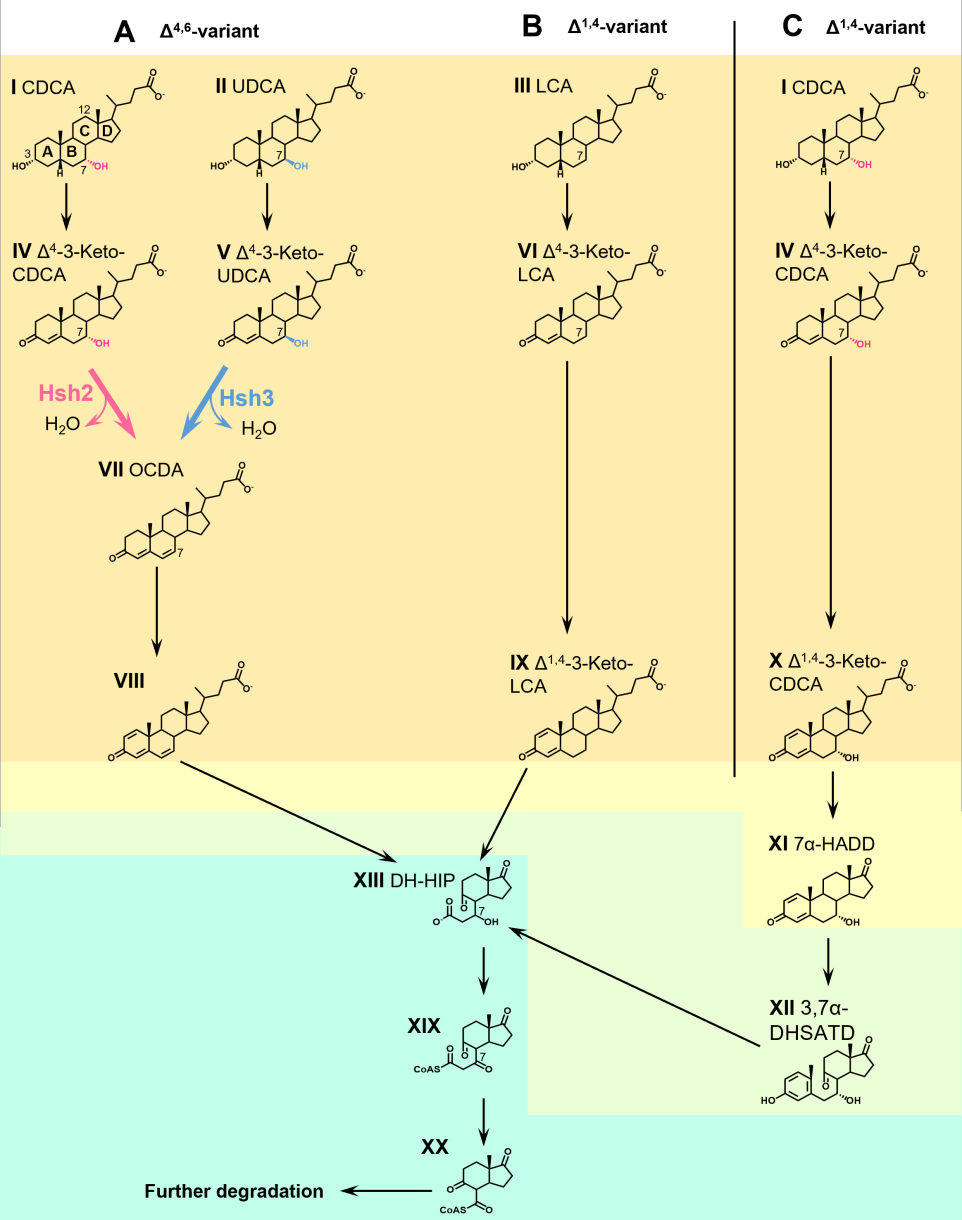
Degradation of bile salts via two variants of the aerobic 9,10-*seco* pathway in *Sphingobium* sp. strain Chol11 (**A**) and *Pseudomonas stutzeri* Chol1 (**B and C**). I, chenodeoxycholate (CDCA); II, ursodeoxycholate (UDCA); III, lithocholate (LCA); IV, Δ^4^-3-keto-CDCA; V, Δ^4^-3-keto-UDCA; VI, Δ^4^-3-keto-LCA; VII, 3-oxo-chol-4,6-diene-oate (OCDA); VIII, 3-oxo-chol-1,4,6-triene-oate; IX, Δ^1,4^-3-keto-LCA, X, Δ^1,4^-3-keto-CDCA; XI, 7α-hydroxy-androsta-1,4-diene-3,17-dione (7α-HADD); XII, 3,7α-dihydroxy-9,10-seco-androsta-1,3,5-triene-9,17-dione (3,7α-DHSATD); XIII, 3′,5-dihydroxy-3aα-*H*-4α(3′-propanoate)−7aβ-methylhexahydro-1,5-indanedione (DH-HIP); XIX, 3′-oxo-5-hydroxy-3aα-*H*-4α(3′-propanoate-CoA)−7aβ-methylhexahydro-1-indanone; XX, 5-hydroxy-3aα-*H*-4α(3′-carboxyl-CoA)−7aβ-methylhexahydro-1-indanone. Red, 7α-hydroxy group and Hsh2-catalyzed reaction; blue, 7β-hydroxy group and Hsh3-catalyzed reaction. Orange shading, A-ring oxidation; yellow shading, side chain degradation; green shading, A- and B-ring degradation; teal shading, HIP degradation.

Bile salts are steroid compounds with different hydroxylation patterns that are produced by vertebrates. The dominant human bile salts all have a 3α-hydroxy group and can have an additional 7α- and 12α-hydroxy groups. In contrast, ursodeoxycholate (UDCA, II in Fig. 1; see Table S1 for overview of all compound abbreviations and molecular masses) is a minor bile salt in humans (1), which has a 3α-hydroxy and a 7β-hydroxy group. It can be found as the dominant bile salt in Asian black bears (2, 3). Furthermore, UDCA is being used to treat different medical conditions (4, 5), especially liver diseases (6, 7) and gallstones (8, 9). For this, large amounts of UDCA (15 mg kg^−1^ body weight day^−1^) are administered, making up most of the bile salt pool during treatment (4, 10, 11) and requiring increasing production of UDCA. Currently, most of the UDCA is produced chemically and is not extracted from black bear bile anymore. New approaches to biotechnological UDCA production are subject to research (12, 13).

While most bile salts are recycled in the so-called enterohepatic cycle, significant amounts are excreted (1, 14, 15). In the environment, bile salts can be degraded by bacteria via pathways similar to the degradation of other steroid compounds. Aerobic bile salt degradation via the 9,10*-seco* pathway has been the subject of intensive research for over 50 years and involves ring cleavage reactions by oxygenases (15, 16) (Fig. 1). One variant of this pathway (Δ^1,4^-variant) can be found in *Pseudomonas stutzeri* Chol1 and *Comamonas testosteroni* and is well elucidated (17, 18) (Fig. 1C). A second variant (Δ^4,6^-variant) can be found in sphingomonads such as *Sphingobium* sp. strain Chol11 and is only partly elucidated (15, 19, 20) (Fig. 1A). Both pathway variants can be structured into four steps: bile salts are first oxidized to Δ^4^-3-keto bile salts (e.g., IV, V, and VI in Fig. 1; step 1: A-ring oxidation, orange in Fig. 1) (20–26). In step 2, side chain degradation yields C19-steroids (yellow in Fig. 1) (15, 27–30). In the Δ^1,4^-variant, androsta-1,4-diene-3,17-diones (ADDs, e.g., XI) are formed, which can be degraded aerobically via the 9,10-*seco* pathway initiated by monooxygenase-mediated cleavage of the B-ring (step 3: degradation of rings A and B, green) (17, 25, 31–34). A- and B-ring are degraded by *meta-*cleavage, hydrolytic and β-oxidation reactions (35–37), leading to derivatives of H-methyl-hexahydro-indanone-3-propenoate (HIP, e.g., XIII). HIPs are central intermediates in both aerobic and anaerobic steroid degradation and are further degraded by β-oxidation and hydrolysis reactions (step 4: HIP degradation, teal) (15, 27, 38, 39). Additional reactions channel the differently hydroxylated bile salts into common pathways. The 12α-hydroxy group is isomerized before ring cleavage reactions and is then removed from HIP compounds (XIII in Fig. 1) by dehydration and reduction reactions (27, 28, 40). In contrast to this, the fate of the 7-hydroxy group differs depending on its conformation and the pathway. A hydroxy group at the position of the former C7 is needed for the β-oxidation-like degradation of the former B-ring of HIP compounds (27). Therefore, a hydroxy group is introduced at this position in HIP if not present in the substrate bile salts by introducing a double bond and hydration in *P. stutzeri* Chol1 and *C. testosteroni* (27, 41). While degradation of 7α-hydroxylated bile salts is well elucidated and found in many microorganisms, only a few organisms have been described to degrade UDCA via this pathway, indicating that the 7β-hydroxy group hinders degradation via this pathway (20, 42).

In the Δ^4,6^-variant, 7-hydroxy bile salt degradation proceeds differently (20): the 7-hydroxy group is eliminated as water, leading to the formation of a double bond in the B-ring in Δ^4,6^-keto compounds, which are then transformed via A-ring oxidation to Δ^1,4,6^-3-keto compounds (e.g., VII). The latter are the substrates for further degradation, including side chain removal via a yet largely unknown reaction sequence and most probably 9,10-*seco* cleavage (31). Genomic and proteomic studies strongly indicate that the degradation of the steroid nucleus is similar to degradation via the well-elucidated 9,10-*seco* pathways as described above and also proceeds via HIP compounds (XIII) (43). For 7α-hydroxy bile salts, the dehydration reaction is catalyzed by 7α-hydroxy steroid dehydratase Hsh2 (20). A similar reaction catalyzed by a homologous enzyme, BaiE, can be found during the transformation of so-called primary 7α-hydroxy bile salts (e.g., chenodeoxycholate, CDCA, I) to secondary 7-deoxy bile salts by *Clostridium* members of the human gut microbiota (e.g., lithocholate, LCA, III) (44, 45). *Sphingobium* sp. strain Chol11 also degrades UDCA via the Δ^4,6^-pathway variant. However, Hsh2 does not catalyze 7β-hydroxy bile salt dehydration, and the deletion mutant still degrades UDCA via Δ^4,6^-3-keto compounds (VII), pointing at a second enzyme for 7β-dehydration (20). The 7β-dehydratase reaction is also found during the transformation of UDCA to LCA by *Clostridia* in the gut during UDCA treatment. This reaction was hypothesized to be catalyzed by BaiI (14), but to the best of our knowledge, its function was not verified so far.

Therefore, our goal was to identify the 7β-hydroxy steroid dehydratase from the UDCA degradation pathway in *Sphingobium* sp. strain Chol11. As no other 7β-hydroxy steroid dehydratases were reported before, we attempted biochemical purification of this enzyme.

## RESULTS

### The 7β-hydroxysteroid dehydratase in *Sphingobium* sp. strain Chol11 is encoded by a second gene and different from 7α-hydroxysteroid dehydratase Hsh2

**Fig 2:**
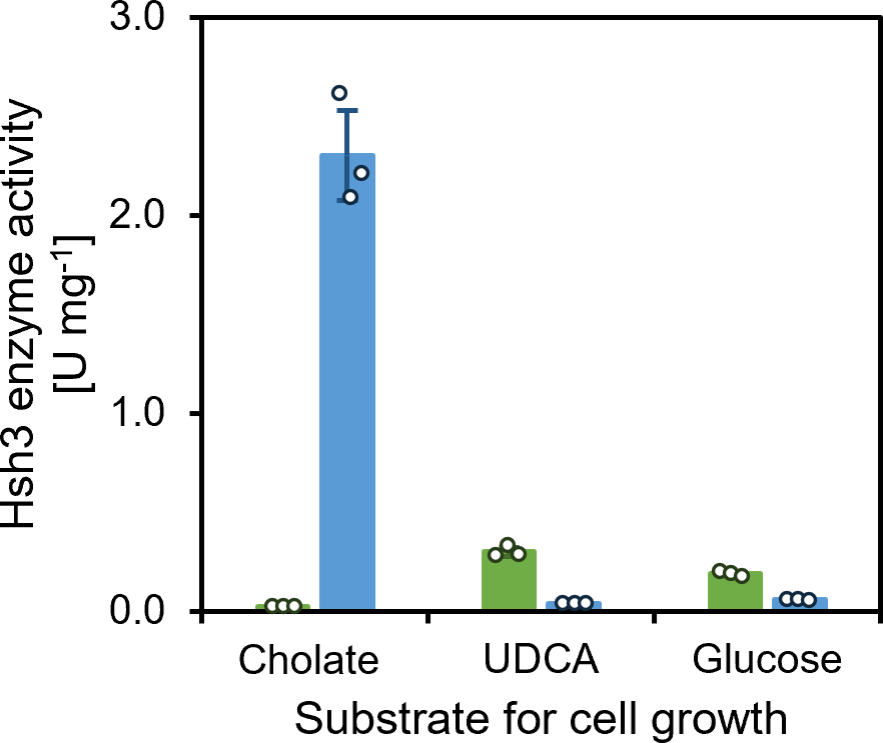
7β-hdroxysteroid dehydratase activities in *Sphingobium* sp. strains Chol11 wt (green) and Δ*hsh2* (blue) cell extracts. Enzyme assays with extracts of cells grown with cholate, UDCA, or glucose as indicated, and Δ^4^-3-keto-UDCA (V in Fig. 1) as substrate. Bars, mean values (*n* = 3); dots, individual data points; error bars, standard deviation of the mean values.

As previously described, bile salt degradation intermediates with a Δ^4,6^-3-keto structure transiently accumulate in cultures of *Sphingobium* sp. strain Chol11 grown not only with the 7α-hydroxy bile salts cholate and CDCA, but also the 7β-hydroxy bile salt UDCA (20). While no Δ^4,6^-3-keto intermediates were formed from cholate and CDCA by strain Chol11 Δ*hsh2,* this strain still produced Δ^4,6^-3-keto intermediates from UDCA. Accordingly, a dehydratase activity toward the 7β-hydroxy substrate Δ^4^-3-keto-UDCA leading to the formation of 3-oxo-chol-4,6-diene-oate (OCDA, VII in Fig. 1) could be observed in cell extracts of *Sphingobium* sp. strain Chol11 as well as in cell extracts of strain Chol11 Δ*hsh2* (Fig. 2 and as confirmed by HPLC-MS for intact cells, not shown), further confirming that this strain contains a second hydroxysteroid dehydratase that is able to transform 7β-hydroxy substrates. Surprisingly, the highest specific 7β-hydroxysteroid dehydratase activity was observed in the cell extracts of *Sphingobium* sp. strain Chol11 Δ*hsh2* grown with cholate, not UDCA. However, bioinformatical prediction and heterologous expression of BaiI homologs in *E. coli* did not lead to any active 7β-hydroxysteroid dehydratase (Text S1; Table S1), indicating the existence of a different type of hydroxysteroid dehydratase.

### The 7β-hydroxysteroid dehydratase from *Sphingobium* sp. strain Chol11 is encoded by *nov2c681*

The same reverse-genetics approach of protein purification and identification by peptide mass fingerprinting, as previously employed for the identification of Hsh2 (20), was used for the identification of the 7β-hydroxysteroid dehydratase. As the highest 7β-hydroxysteroid dehydratase activity was found in the cell extracts of *Sphingobium* sp. strain Chol11 Δ*hsh2*, further protein purification was performed using cell extracts from this strain. An enzyme assay using Δ^4^-3-keto-UDCA (V in Fig. 1, absorption maximum 245 nm) as substrate was used for all purification steps as activity could easily be monitored spectrophotometrically by the formation of the product OCDA (VII, absorption maximum at 290 nm). Final purification after ammonium sulfate precipitation and anion exchange chromatography was achieved by native PAGE (Fig. S1), from which bands were excised and tested for 7β-hydroxysteroid dehydratase activity. The active band was subjected to peptide mass fingerprinting and identified as Nov2c681 (RefSeq ID WP_097093216.1).

**Fig 3:**
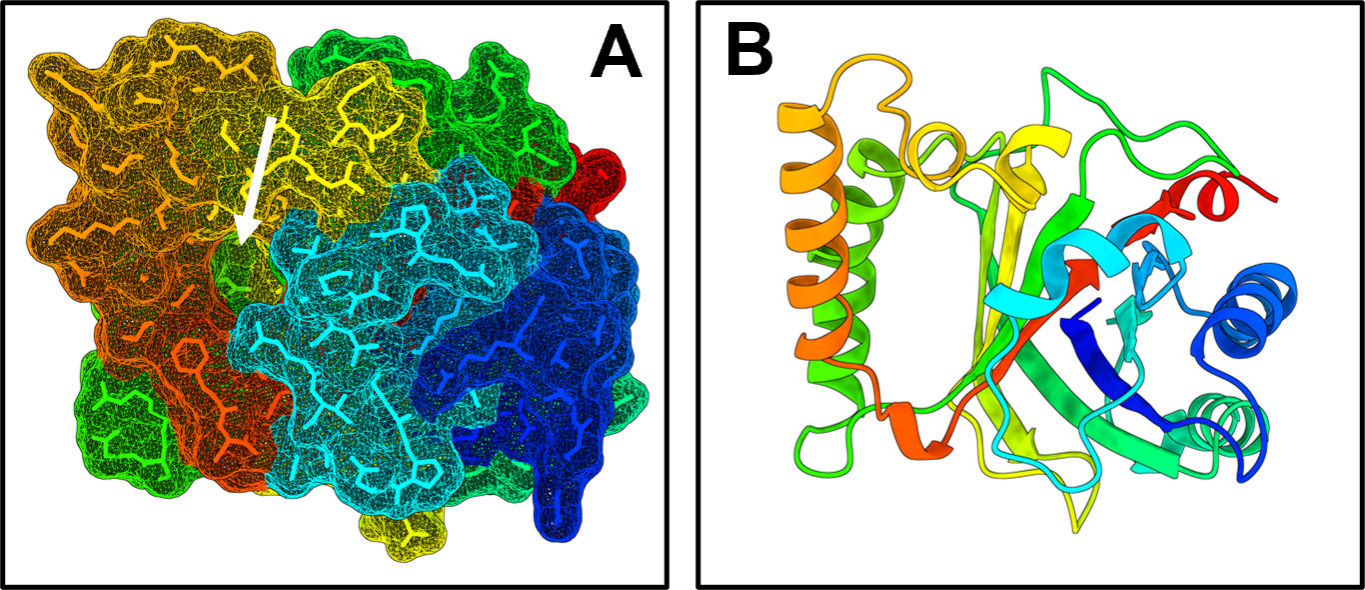
AlphaFold3 prediction of the structure of Hsh3 from *Sphingobium* sp. strain Chol11. (**A**) All-atom representation with the molecular surface. The white arrow indicates a cavity. (**B**) Ribbon diagram from the same angle as (**A**). Confidence scores for most of the structures were >90 (Fig. S2). The structure is colored in a rainbow gradient by residue (re, C-terminus; blue, N-terminus) to enhance clarity.

In contrast to 7β-hydroxysteroid dehydratases Hsh2 and BaiE, Nov2c681 does not belong to the NTF2-like superfamily but has an EthD domain and belongs to the dimeric α/β barrel proteins. Accordingly, it only has 16% and 12% identity with Hsh2 and BaiE from *C. scindens* VPI12708. It also has a low similarity (12% identity) to the putative 7β-hydroxy bile salt dehydratase BaiI from *C. scindens* ATCC35704. An AlphaFold3 prediction of the structure of Hsh3 indicated the presence of eight α-helices, which are organized in a nearly mirror-symmetrical manner around a barrel-like core of nine β-sheets (Fig. 3; Fig. S2). Overall, one major cavity was predicted (Fig. 3A), which might represent an active site.

### Nov2c681/Hsh3 catalyzes the 7β-dehydration of UDCA-derived compounds

For testing the activity of Nov2c681, *nov2c681* was heterologously expressed in *P. stutzeri* Chol1, resulting in 7β-hydroxysteroid dehydratase activity (Fig. S3). While *Sphingobium* sp. strain Chol11 and *P. stutzeri* Chol1 use different pathway variants for the degradation of bile salts, *P. stutzeri* also transiently accumulates steroid intermediates such as Δ^4^-3-keto derivatives that might be the substrates of Nov2c681 and can therefore be used to test the activity of bile salt degradation enzymes with different intermediates. *P. stutzeri* Chol1 pBBR1MCS-5::*nov2c681* transformed UDCA (II in Fig. 1) to 3-hydroxy-9,10-*seco*-androsta-1,3,5(10),6-tetraene-9,17-dione (HSATD, XXI, Fig. S2) with a Δ^6^ double bond instead of 3,7β-dihydroxy-9,10-*seco*-androsta-1,3,5(10)-triene-9,17-dione (3,7β-DHSATD) that was produced by the wildtype strain (20). The production of HSATD also resulted in the formation of a purple pigment that precipitated and could not be identified further, similar to the formation of a purple pigment from 3,12β-dihydroxy-9,10-*seco*-androsta-1,3,5(10),6-tetraene-9,17-dione (3,12β-DHSATD) at basic pH as reported before (46). Additionally, the enzyme was tested in *P. stutzeri* Chol1 Δ*kstD1* Δ*scdA1. P. stutzeri* Chol1 Δ*kstD1* Δ*scdA1* lacks the coenzyme A ligase that initiates degradation of the side chain as well as the ketosteroid dehydrogenase that is needed for further degradation of the steroid nucleus and therefore transforms bile salts to their corresponding Δ^4^-3-keto derivatives (26). *P. stutzeri* Chol1 Δ*kstD1* Δ*scdA1* pBBR1MCS-5::*nov2c681* produced OCDA (VII) from UDCA, while CDCA (I) was transformed to Δ^4^-3-keto-chenodeoxycholate (IV) (Fig. S2). Thus, Nov2c681 has specific 7β-hydroxysteroid dehydratase activity when produced in strain Chol1, and it was renamed Hsh3 for hydroxysteroid dehydratase 3.

### Hsh3 is active with different 7β-hydroxy steroids as well as minor activity with 7α-hydroxy bile salts

**Fig 4:**
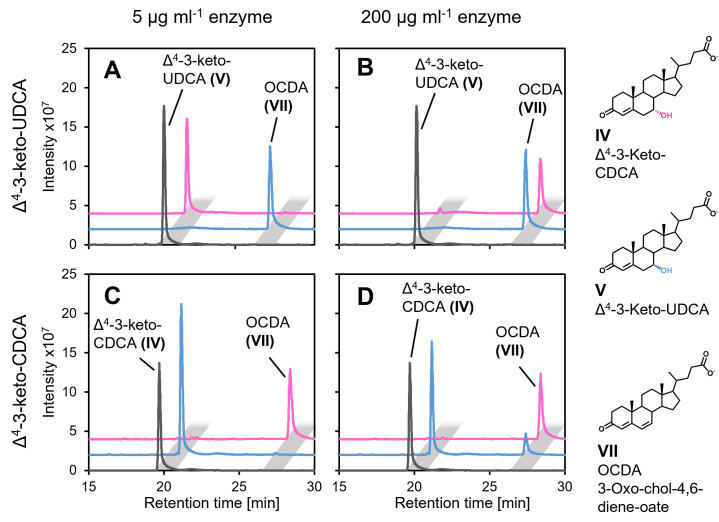
Transformation of Δ^4^-3-keto-UDCA (A and B) and Δ^4^-3-keto-CDCA (C and D) by purified Hsh3 (blue) and Hsh2 (red) from *Sphingobium* sp. strain Chol11 added in different concentrations (A and C, 5 µg mL^−1^; B and D, 200 µg mL^−1^) compared to a control without enzyme (gray). Incubated for 30 min at 30°C. MS base peak chromatograms in negative mode with offset in intensity and retention time for more clarity. Light gray rectangles indicate peaks with the same retention time.

For characterizing Hsh3, especially in comparison with Hsh2, which has a very similar function, both enzymes were purified using a His-tag and affinity chromatography as well as gel filtration (Fig. S4). Enzyme assays confirmed that Hsh3 is able to catalyze the dehydration of Δ^4^-3-keto-UDCA (V) to OCDA (VII), and we observed the complete transformation of 1 mM Δ^4^-3-keto-UDCA to OCDA by 5 µg mL^−1^ Hsh3 within 30 min (Fig. 4A).

In contrast to this, the kinetic parameters of the two enzymes were not determined in enzyme assays with Δ^4^-3-keto bile salts. Instead, the side chain-less compounds 7α- or 7β-hydroxy ADD (7α-HADD, XI in Fig. 1; 7β-HADD, XXII in Fig. S5) for Hsh2 or Hsh3, respectively, were used, which were also transformed by these enzymes and allowed for more precise determination of transformation rates due to their absorption maximum at 300 nm (Fig. S5; Table 1). The *k*_cat_ of Hsh3 was about eight times as high as the *k*_cat_ of Hsh2, and Hsh3 had about fivefold higher *K*_M_ compared to Hsh2.

**TABLE 1:** Kinetic parameters of purified Hsh2 and Hsh3 from *Sphingobium* sp. strain Chol11 as determined with 7α-HADD and 7β-HADD, respectively, as mentioned in the table

Enzyme	*K*_M_ (mM)	*k*_cat_ [s^−1^)	*k*_cat_/K_M_ (mM^−1^ s^−1^)	Specific activity at [*S*] = 1 mM (U mg^−1^)
Hsh3, with 7β-HADD	1.26 ± 0.45	129.67 ± 30.10	102.91	130.311
Hsh2, with 7α-HADD	0.25 ± 0.05	15.63 ± 0.0008	62.52	38.13

For further characterization, the ability to transform substrates with the respective other 7-hydroxy group was tested. When only 5 µg mL ^−1^ enzyme was added to the assay, only the respective substrates were transformed (Fig. 4A and C). However, the addition of much higher enzyme concentrations of 200 µg mL^−1^ led to nearly complete transformation of Δ^4^-3-keto-UDCA (IV) after 30 min also by Hsh2, and limited transformation of Δ^4^-3-keto-CDCA (V) by Hsh3 (Fig. 4B and D).

### Hsh3 is involved in the degradation of 7β-hydroxy bile salt UDCA in *Sphingobium* sp. strain Chol11 but not essential

**Fig 5:**
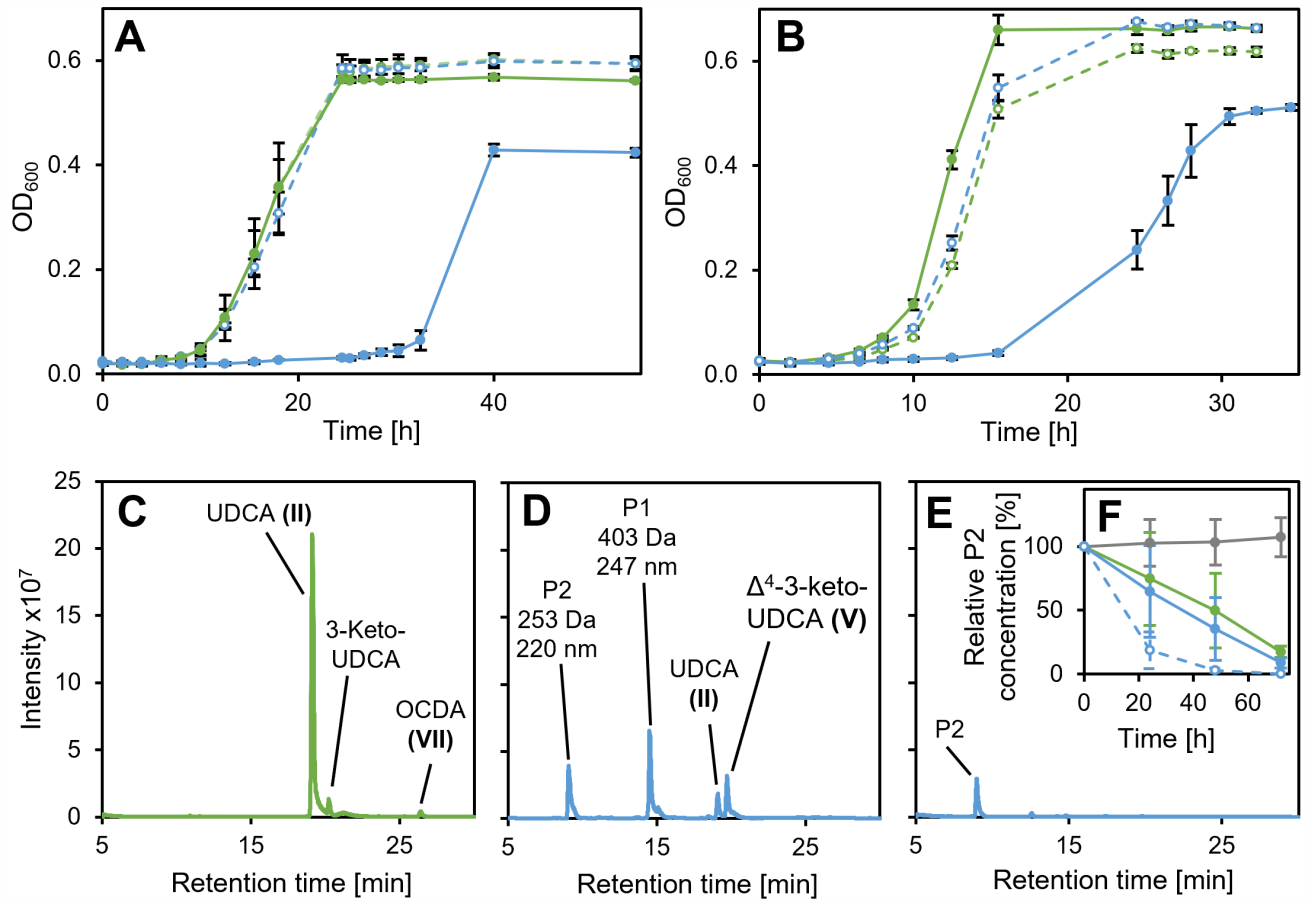
Growth of *Sphingobium* sp. strains Chol11 wt and Chol11 Δ*hsh3* with UDCA. (**A**) Growth of strain Chol11 Δ*hsh3* (blue) and wild type (green) with either UDCA (solid lines) or CDCA (dashed lines). (**B**) Growth of strain Chol11 Δ*hsh3* (blue) and wt (green) with pBBR1MCS-5 (solid lines) or with pBBR1MCS-5::*hsh3* (dashed lines) with UDCA. (**C**) Transient accumulation of Δ^4,6^-3-keto compound OCDA during degradation of UDCA by strain Chol11 wildtype. (**D**) Accumulation of Δ^4^-3-keto UDCA as well as two new compounds (P1 and P2) by strain Chol11 Δ*hsh3* during degradation of UDCA. (**E**) Accumulation of product P2 in cultures of strain Chol11 Δ*hsh3* in stationary phase after 26 h. Supernatants from exponential phase (**C and D**) or stationary phase (**E**) cultures. For (C –E), MS base peak chromatograms in negative mode are shown. Compounds are identified by *m/z*, absorption spectrum, and retention time. (**F**) Degradation of P2 (100% = initial concentration) by cell suspensions of strain Chol11 wt (green, OD_600_ = 1) or Δ*hsh3* (blue; solid lines, OD_600_ = 1; dashed, OD_600_ = 2) in comparison to a sterile control (gray). Cell suspensions were prepared from actively growing cultures of the respective strain and incubated at 30°C after the addition of P2 isolated from another culture. All error bars indicate standard deviation of mean values (*n* = 3).

For confirming the function of Hsh3 in UDCA degradation of *Sphingobium* sp. strain Chol11, the deletion mutant *Sphingobium* sp. strain Chol11 Δ*hsh3* was constructed. *Sphingobium* sp. strain Chol11 Δ*hsh3* displayed delayed growth and lower yields during growth with UDCA compared to growth with other bile salts. The lag phase increased to up to 30 h with UDCA, while no detectable lag phase was found with other bile salts, and the final OD_600_ was about 15% to 30% lower than with other bile salts (Fig. 5A). Plasmid-based expression of *hsh3* in the deletion mutant reversed the phenotype (Fig. 5B). Expression of *hsh2* did not lead to wt-like growth of *Sphingobium* sp. strain Chol11 Δ*hsh3* (not shown).

### *Sphingobium* sp. strain Chol11 Δ*hsh3* produces a HIP compound that can only slowly be degraded

**Fig 6:**
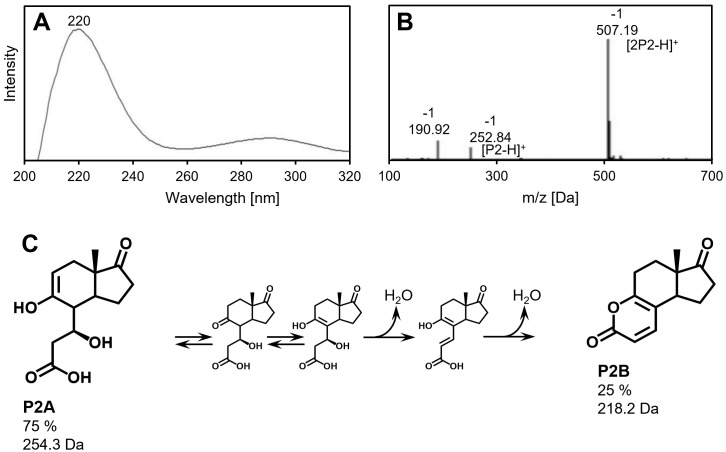
Characteristics and structure of compound P2 produced by *Sphingobium* sp. strain Chol11 Δ*hsh3* from UDCA. (**A**) UV spectrum of P2. (**B**) Mass spectrum of P2. (**C**) Structures of the compounds P2A and P2B found in the extracted P2 solution including a potential mechanism for the abiotic formation of P2B from P2A.

During growth with UDCA, *Sphingobium* sp. strain Chol11 Δ*hsh3* accumulated several additional compounds compared to the wildtype, but no Δ^4,6^-3-keto (e.g., VII in Fig. 1) compounds as indicated by the respective UV spectra, which can give additional information about the affected degradation pathway (Fig. 5D). During the exponential growth phase, UDCA (II) as well as Δ^4^-3-keto-UDCA (V) was found. Additionally, one so-far unknown compound P1 with a mass of 403 Da and an absorption maximum at 247 nm was observed (Fig. S6). The absorption spectrum indicates a Δ^4^- or Δ^1,4^-3-keto structure of the steroid skeleton. Interestingly, the 16 Da higher mass compared to Δ^4^-3-keto-UDCA indicates a hydroxylation at a so-far unknown position. Another compound, P2, was present in these cultures and remained in the supernatant even after several days of incubation (Fig. 5E). This compound had a molecular mass of 253 Da and an absorption maximum at 220 nm (Fig. 6A and B). The culture of the deletion mutant was slightly more yellow compared to the wild-type cultures, which was not due to cell color but the supernatant (Fig. S7A). To determine if P2 could be a natural degradation intermediate of *Sphingobium* sp. strain Chol11 or a modified side product, degradation of the compound by strain Chol11 was tested. Interestingly, both strain Chol11 wt and strain Chol11 Δ*hsh3* were able to degrade over 80% of the added amount within several days (Fig. 5F). Biotic degradation was confirmed by a sterile control, in which no degradation was observed, and a control containing two times as many cells, in which degradation was about two times as fast. The mass of compound P2 indicated a structure similar to HIP compounds (e.g., XIII). For analyzing P2 in more detail, it was purified by extraction, which resulted in a yellow solution (Fig. S7B), and HPLC purification. P2 was acid- but not base-stable (Fig. S7C through E). The structure of P2 was analyzed using NMR measurements (Fig. S8). Although P2 was purified using HPLC, two distinct structures P2A and P2B with different molecular masses were identified by NMR (Fig. 6C). The mixture contained 75% compound P2A and 25% compound P2B. As predicted, P2A is a HIP compound, 3′,5-dihydroxy-H-methyl-hexahydro-5-indene-1-one-propanoate (3,5-DH-HIEP), which still has the former 7β-hydroxy group. In addition, it has a double bond in the former C-ring, and the former 9-keto group is reduced to a hydroxy group. The second product P2B has a lactone ring with two double bonds in the former C-ring.

### Hsh3 homologs and activity can be found in several bile salt-degrading *Sphingomonadaceae* strains, further expanding the metabolic repertoire for bile salt in these strains

**Fig 7:**
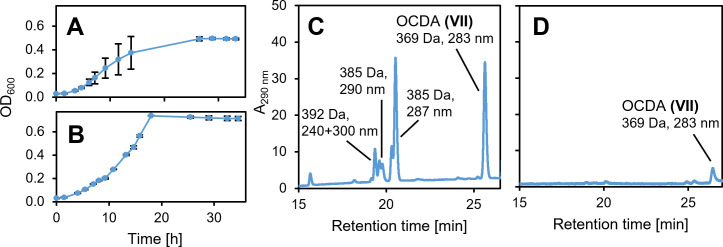
Growth of other sphingomonads with UDCA and accumulation of Δ^4,6^-3-keto compounds. (**A and C**) *Novosphingobium aromaticivorans* F199. (**B and D**) *Caenibius tardaugens* NBRC16725. (**A and B**) Growth with 1 mM UDCA. (**C and D**) MS base peak chromatograms in negative mode. All error bars indicate standard deviation of mean values (*n* = 3).

Recently, bile salt degradation via the Δ^4,6^-pathway was predicted for many strains of the *Sphingomonadaceae* and confirmed for three of these strains, *Sphingobium herbicidovorans* MH, *Caenibius tardaugens* NBRC16725, and *Novosphingobium aromaticivorans* F199 (43). A reciprocal BLASTp analysis showed that all three strains contain one single homolog of Hsh3 with very high identities of at least 62% to Hsh3 (Table 2). Growth experiments confirmed the growth of *N. aromaticivorans* F199 and *C. tardaugens* NBRC16725 with UDCA and a transient accumulation of Δ^4,6^-intermediates during growth (Fig. 7).

**TABLE 2:** Hsh3 homologs in other bile salt-degrading strains of the *Sphingomonadaceae* as determined by BLASTp analysis^*a*^

Strain	Hsh3 homolog	Identity	*E* value
*S. herbicidovorans* MH	WP_234810817.1	84%	1e−117
*N. aromaticivorans* F199	WP_011906908.1	62%	6e−101
*C. tardaugens* NBRC16725	WP_021690432.1	64%	2e−112

^*a*^ RefSeq accession numbers are given as identifiers.

### UDCA-degrading bacteria are present in a wide variety of environments with bile salt input

Using an HMM search on the MGnify website revealed homologs for Hsh3 as well as Hsh2 could be found in metagenomes from many environments, especially from environments classified as environmental—marine and engineered—wastewater (Fig. S9). The highest fraction of assemblies with Hsh3 and Hsh2 homologs was from aquaculture environments. Strikingly, only very few homologs were found in metagenomes from host-associated environments, and none in metagenomes from human digestive systems.

For finding further UDCA degraders and finding potential other pathways for UDCA degradation, enrichment cultures with UDCA were inoculated from different sites with bile salt input such as duck ponds and soil close to agricultural fields. This way, many UDCA-degrading strains could be isolated, pointing to a wide distribution of this capacity in the environment. According to their 16S rRNA sequences, some strains belonged to the *Sphingomonadaceae,* but most strains belong to the groups *Pseudomonas* or *Comamonas* (Fig. S10). As expected, the *Sphingomonadaceae* strains transiently accumulated Δ^4,6^-intermediates, but some *Pseudomonas* and *Comamonas* strains were also able to completely degrade UDCA without transient accumulation of Δ^4,6^ compounds and only produced Δ^1,4^ compounds indicating the existence of an additional pathway (Fig. S10).

## DISCUSSION

With Hsh3, we found an additional enzyme modifying the diverse hydroxy groups of bile salts and described the first 7β-hydroxysteroid dehydratase. The different hydroxylation patterns of bile salts among different animals lead to an astonishing diversity of these compounds (47). At the same time, many bacteria interacting with these compounds have evolved enzymes to modify bile salts at their hydroxy groups. On the one hand, intestinal bacteria oxidize and eliminate these hydroxy groups as well as change their conformation (48). On the other hand, environmental bacteria degrade bile salts as energy and carbon sources and modify the hydroxy groups to allow degradation via unified pathways (15). Our data show that 7β-hydroxysteroid dehydratase Hsh3 catalyzes the dehydration of the 7β-hydroxy group of UDCA, thus allowing *Sphingobium* sp. strain Chol11 to channel this bile salt into the same pathway as CDCA and CA, which are dehydrated by the 7α-counterpart Hsh2 (20). Its homologs in various sphingomonads add to the wide bile salt substrate spectrum of these bacteria (43). UDCA-degrading strains transiently accumulating Δ^4,6^-steroid intermediates could also be isolated from lakes and agricultural soils in Germany. Additionally, we found homologs of Hsh3 in many wastewater treatment plants and aquatic microbiomes, especially aquaculture microbiomes, which are probably also contaminated with bile salts. Taken together, this indicates a wide distribution of the Δ^4,6^-degradation pathway and points to the widespread presence of 7β-hydroxysteroid compounds in these environments.

In previously described proteome analyses of *Sphingobium* sp. strain Chol11 (43), Hsh3 was found in the proteomes of cells grown with all tested substrates cholate, deoxycholate, 7α,12β-dihydroxy ADD (7α,12β-DHADD), and glucose with higher amounts in the steroid-grown cells compared to the glucose control. Similar to 7α-hydroxysteroid dehydratase Hsh2, Hsh3 is encoded on chromosome 2 of strain Chol11, which harbors the steroid degradation genes, in vicinity to genes for hydroxysteroid dehydrogenases but not within the main steroid degradation clusters (15, 49)

Despite its role in UDCA degradation in *Sphingobium* sp. strain Chol11, Hsh3 does not seem to be indispensable for UDCA degradation, with deletion of *hsh3* only leading to delayed growth and decreased yields. As expected, no Δ^4,6^-intermediates were observed in the supernatants of UDCA cultures. This is similar to *Sphingobium* sp. strain Chol11 Δ*hsh2*, which is also able to grow with 7α-hydroxy bile salts without forming Δ^4,6^-intermediates (20). However, *Sphingobium* sp. strain Chol11 Δ*hsh3* accumulates two new intermediates when grown with UDCA, which highlights bottlenecks in degradation by this strain and might explain the lower final OD_600_. Intermediate P1, which is only transiently accumulated, has a Δ^1,4^- or Δ^4^-3-keto structure, like the other intermediates found in this culture. However, the mass of P1 indicates one additional hydroxylation compared to UDCA, the position of which is unclear. Other hydroxylated compounds have been found in the supernatants of *Sphingobium* sp. strain Chol11 Δ*sclA* and Δ*scd4A*, which carry deletions of side chain degradation genes (31). On the one hand, the hydroxylation could be at C9, a reaction necessary for later B-ring cleavage, which is catalyzed by KshA homologs Nov2c407, Nov2c430, and Nov2c440 in *Sphingobium* sp. strain Chol11. On the other hand, it might be in another position and important for side-chain degradation. This hydroxylation is probably catalyzed by the monooxygenase Nov2c228 in *Sphingobium* sp. strain Chol11 (31). As P1 and other intermediates with side chains are subsequently degraded by *Sphingobium* sp. strain Chol11 Δ*hsh3*, the β-hydroxy group seems to slow down degradation during or after side chain degradation. The other new intermediate, P2A, remained in the culture supernatant after the culture reached stationary phase but could be degraded by both the wildtype strain and *Sphingobium* sp. strain Chol11 Δ*hsh3*. By NMR spectroscopy, P2A was shown to be a HIP compound consisting of only the C- and D-ring with a C_5_-side chain from the former B-ring. As expected, the former 7β-hydroxy group is still found in P2A. An unusual enol structure, which probably is a result of keto-enol tautomerism, is most probably stabilized by an intramolecular hydrogen bond between the enol hydroxy group and the carboxy group, as indicated by NMR spectroscopy (Fig. S11). A second structure P2B found together with P2A after purification could be derived from P2A abiotically by dehydration, which has also been observed for steroid compounds under acidic conditions as used for extraction (50) and lactone formation, which has also been observed before for these HIP compounds (51, 52).

For *C. testosteroni*, it was suggested that degradation of HIP compounds from steroids involves a β-hydroxy group in the former C7 position. During the degradation of 7α-hydroxy steroids such as cholate and CDCA, this would be achieved by consecutive dehydration (similar to Hsh2 reaction, but later in degradation) and hydration in β-conformation (53). Therefore, it might be speculated that the Hsh2 reaction in sphingomonads is a preparation for this step, especially as this reaction can be observed as a minor—potentially side—reaction in *C. testosteroni* as well (40). However, degradation of the β-hydroxy HIP (P2A) was strongly delayed in *Sphingobium* sp. strain Chol11 Δ*hsh3*. Additionally, *Sphingobium* sp. strain Chol11 Δ*hsh2* did not accumulate similar HIP compounds as persistent intermediates. This indicates that, at least in *Sphingobium* sp. strain Chol11*,* a 7α-hydroxy group is needed for β-oxidation of the HIP side chain. In either case, the hydroxy group is first eliminated by Hsh2 or Hsh3, and then the hydroxy group in a suitable conformation is added at a later stage. This could indicate either a strategy to funnel all bile salts into the same pathway or point at enzymes that could be inhibited by these hydroxy groups.

Hsh3 and Hsh2 displayed minimal activity with the respective other substrate (Δ^4^-3-keto-UDCA (V) for Hsh2 and Δ^4^-3-keto-CDCA (IV) for Hsh3). However, this minimal activity was not enough to reverse the phenotype of *Sphingobium* sp. strain Chol11 Δ*hsh3*, even if *hsh2* was overexpressed from an expression vector. At the same time, the side activity of the two enzymes could explain the high Hsh3 activity in the *Sphingobium* sp. strain Chol11 Δ*hsh2* cell extract: *hsh3* might be overexpressed to compensate for the *hsh2* deletion.

Interestingly, Hsh3 is very different from Hsh2 despite catalyzing very similar reactions, indicating different evolutionary origins. Additionally, Hsh3 is structurally different from the proposed 7β-hydroxysteroid dehydratase BaiI from *C. scindens,* and Hsh3 homologs are not present in *C. scindens*. This points to the existence of phylogenetically distant additional 7β-hydroxy dehydratases in *Clostridia,* which is further emphasized by the lack of Hsh3 homologs in *Clostridium* genomes according to our BLAST search. Additionally, we did not find any Hsh3 homologs in human digestive system metagenomes in the MGnify database or in the metagenomes of the human microbiome project (54). However, we also did not find any Hsh2 homologs in these metagenomes, despite the high similarity of Hsh2 and BaiE, which indicates that our analysis might be too specific to find any more distant homologs in metagenomic data sets.

Besides its relevance for the degradation of UDCA, which is probably released into the environment in large amounts due to its pharmaceutical use, Hsh3 could be of interest for biotechnological applications for the production of tailored bile salts. Hydroxysteroid dehydratases Hsh2 and Hsh1 are similar enzymes but catalyze the elimination and addition of hydroxy groups in steroidal compounds, respectively. Therefore, a reverse Hsh3 reaction producing UDCA-like compounds seems possible and interesting.

## MATERIALS AND METHODS

### Bacterial strains and cultivation

Bacterial strains were cultivated as described previously (31), and strains and plasmids used in this study are listed in Table 3. Strains of *E. coli* were grown in lysogeny broth medium (LB [55]) if not described otherwise. Strains of *Sphingobium* sp. strain Chol11 and *P. stutzeri* Chol1 were grown in the HEPES buffered medium B (MB [56]) with 1 mM bile salts, 12 mM succinate (strain Chol1), or 15 mM glucose (strain Chol11) as described. *Novosphingobium aromaticivorans* F199 and *Caenibius tardaugens* NBRC16725 were cultivated in MB with 1 mM bile salt as carbon source. *E. coli* strains were cultivated at 37°C on agar plates, and all other strains and *E. coli* strains in liquid cultures were cultivated at 30°C.

**TABLE 3:** Strains and plasmids used in this study^*a*^

Strain or plasmid	Description	Reference
Strains		
*Sphingobium* sp. strain Chol11	Bile-salt degrading strain	DSM 110934 (19)
Strain Chol11 Δ*hsh3*	Deletion mutant of *hsh3*	This study
*Novosphingobium aromaticivorans* F199	Bile-salt degrading strain	DSM 12444 (57)
*Sphingobium herbicidovorans* MH	Bile-salt degrading strain	DSM 11019 (58)
*Caenibius tardaugens* NBRC16725	Bile-salt degrading strain	DSM 16702 (59)
*Pseudomonas stutzeri* Chol1	Bile-salt degrading strain	DSM 103613 (17)
Strain Chol1 Δ*stdA1* Δ*kstD1*	Deletion mutant of *stdA1* and *kstD1*, produces Δ^4^-3-keto bile salts	(26)
*Escherichia coli* MG1655	Expression and testing of *hsh3*, prototrophic, F^−^, lambda^−^ , rph-1	DSM18039 (60)
*Escherichia coli* ST18	Cloning strain, 5-Ala auxotrophic, *pro thi hsdR* + Tpr Smr; chromosome::RP4-2 Tc::Mu-Kan::Tn7/λpir Δ*hemA*	DSM 22074 (61)
*Escherichia coli* Tuner(DE3)	Protein production, *ompT^-^ hsdS*_B_ (r_B_^−^ m_B_^−^) *gal dcm lacY1*(DE3)	Novagen (Merck, Darmstadt, Germany)
Plasmids		
pBBR1MCS-5	Expression vector, Gn^R^	(62)
pET28B(+)	Expression vector, Kn^R^	Novagen (Merck, Darmstadt, Germany)
pDM4	Gene replacement vector, Cm^R^, *sacB*	(63)

^*a*^ Gn^R^, Kn^R^, and CM^R^: resistance against gentamicin, kanamycin, and chloramphenicol, respectively.

For strains containing pBBR1MCS-5, 20 µg mL^−1^ gentamicin was added. For strains containing pDM4 30 (*E. coli*) up to 90 (strain Chol11) µg/mL^−1^ chloramphenicol was added, and for strains containing pET28B(+) 50 µg mL^−1^ kanamycin was added. For the cultivation of *E. coli* ST18, 50 µg mL^−1^ 5-aminolevulinic acid was added. For agar plates, 1.5% bacto agar (BD, Sparks, USA) was added to the respective media.

For pre-cultures, 5 mL medium in test tubes was inoculated from agar plates and incubated at 30°C and 200 rpm overnight (ca. 14 h). For growth experiments, 5 mL cultures in test tubes were inoculated at an initial optical density at 600 nm (OD_600_) of about 0.02 using pre-cultures and incubated at the same conditions. Growth was tracked by measuring OD_600_. HPLC-MS samples were withdrawn at regular intervals, and supernatants were either analyzed directly or stored at −20°C.

### Biotransformation experiments

For transformation of steroid compounds with different bacteria expressing *hsh3* and the respective empty vector controls, cells from pre-cultures were washed and resuspended in MB medium at the given OD_600_. Steroids as well as the respective non-steroid carbon source were added to these cell suspensions before incubation at 30°C. Samples for HPLC-MS measurements were withdrawn after addition of the steroid substrate as well as at further time points, stored at −20°C, and centrifuged prior to HPLC-MS analysis of the supernatant.

### Enrichment, isolation, and characterization of UDCA-degrading strains

Enrichment cultures for the isolation of UDCA-degrading strains were prepared in MB as described (19) and inoculated from environmental samples. Sampling sites included duck ponds, lake Aasee, as well as soil and water from drainage of agricultural fields in the Münsterland area.

A total of 1 mL of water samples was combined with 4 ml MB with 1 mM UDCA and incubated in test tubes at 30°C and 200 rpm. For soil samples, 1 g soil was suspended in 5 ml MB at room temperature and 200 rpm for 1 h. A total of 1 mL of these suspensions was added to 4 ml MB with 1 mM UDCA and diluted 1:10 in the same medium before incubation at 30°C and 200 rpm. Growth and activity in these cultures were tracked by OD_600_ and HPLC-MS measurements of the supernatant. Turbid cultures were transferred to fresh medium three times before being transferred to agar MB agar plates with 1 mM UDCA. Single colonies with different morphology were transferred to new plates three times for isolation of single strains.

For identification of isolates, genomic DNA was extracted from the cells, and the 16S rRNA gene was sequenced after amplification. Strains were then identified using BLASTn (64).

### Cloning techniques and construction of an unmarked deletion mutant

Cloning was performed according to standard procedures and as described (26). Primers for the cloning procedures are listed in Table 4.

**TABLE 4:** Primers used in this study

Name	Sequence
*hsh3*_up_for	TTTTTTTCTCGAGACTCAACTCCCGCATCTTGG
*hsh3*_up_rev	GATATCGGGAGGGGGCTAAG
*hsh3*_dn_for	CTTAGCCCCCTCCCGATATCTCAGATCTCTCCATCAATCT
*hsh3*_dn_rev	TTTTTTTTCTAGACTGATCAACAATGCGCAGG
*hsh3*_exp_for	tttttttctagaCAGAACAGAATGGGAGGAGG
*hsh3*_exp_rev	ttttttctcgagAAGATTGATGGAGAGATCTGA
*hsh3*_pur_rev	TTTTTTCCATGGAAAAGCTGATCTGCGCTCTT
*hsh3*_pur_rev	TTTTTTAAGCTTCTTCCCGTCGAGATGGGCGA
M13 for (−43)	AGGGTTTTCCCAGTCACGACGTT
M13 rev (−49)	GAGCGGATAACAATTTCACACAGG
T7_for	TAATACGACTCACTATAGGG
T7_rev	TATGCTAGTTATTGCTCAG
pDM4_MCS_for	ACTTAACGGCTGACATGGGA
pDM4_MCS_rev	GCGAAGTGATCTTCCGTCAC
pDM4_backbone_for	AAG ATG TGG CGT GTT ACG GT
pDM4_backbone_rev	AGG CTC TGG GAG GCA GAA TA

For expression of proteins in different strains, the respective gene was amplified from genomic DNA using the exp_for and exp_rev primers. The gene and expression plasmid pBBR1MCS-5 were processed using the given restriction enzymes (Table 4), ligated, and transferred into *E. coli* MG1655 or ST18 by heat shock transformation. Plasmids were transferred into strains Chol1 or Chol11 by bi-parental conjugation from *E. coli* ST18. Correct assembly of vectors and transformation were confirmed by colony PCRs using primers M13 and sequencing.

For the construction of deletion mutants, up- and downstream fragments of the respective gene were amplified using primer pairs up_for/rev and dn_for/rev, respectively. The fragments were fused using splicing by overlapping extension (SOE)-PCR (65) using the primer pair up_for/dn_rev and cloned into vector pDM4 using the given restriction enzymes. Correct vector assembly was confirmed using primer pair pDM4_MCS_for/rev after transformation into *E. coli* ST18. The resulting vector was transferred into strain Chol11 by conjugation, and successful conjugation and insertion were confirmed by colony PCR using primer pair pDM4_backbone_for/rev. Unmarked gene deletion after forcing vector excision by cultivation on plates containing 10% sucrose was confirmed by colony PCR with primer pair up_for/dn_rev and sequencing.

### Enrichment, isolation, and identification of Hsh3

Hsh3 was purified from cell extracts of UDCA-grown cells of *Sphingobium* sp. strain Chol11 as described for Hsh2 by reference 20.

For this, four 500 mL cultures of strain Chol11 Δ*hsh2* in MB with 1 mM cholate in Erlenmeyer flasks were inoculated from pre-cultures. Cells of each culture were harvested in the exponential growth phase by centrifugation at 4°C and 4,000 *× g* for 30 min, washed with 50 mM MOPS buffer (pH 7.8), and resuspended in 4 mL 50 mM MOPS buffer (pH 7.8). Cells were then lysed by ultrasonication (UP200S, Hielscher Ultrasonics, Teltow, Germany) with amplitude 60% and cycle 0.5 for 14 min on ice with 1 min breaks every 4 min. Cell debris was removed by centrifugation at 4°C and 25,000 × *g* for 30 min, and cell-free extracts from all cultures were combined for further isolation steps.

The first purification step was ammonium sulfate precipitation. After prior optimization, 40% (wt/vol) ammonium sulfate was added to the cell extracts in a first step. Precipitated proteins were removed by centrifugation at 0°C and 25,000 × *g* for 12 min. In a second step, the ammonium sulfate concentration in the remaining supernatant was increased to 60%, and precipitated proteins were again removed by centrifugation. The supernatant was discarded, and the precipitate of the second step was used for further purification. For this, the precipitate was solved in 50 mM MOPS buffer (pH 7.8) and desalted by gel filtration using PD-10 desalting columns (GE Healthcare, Chicago, IL, USA) as recommended. For equilibration and elution, 3.5 mL 20 mM MOPS buffer (pH 7.0) was used. The resulting protein solution was further separated by anion exchange chromatography using a SOURCE 15Q column (6 mL, GE Healthcare, Chicago, IL, USA). After equilibration with 20 mM MOPS buffer (pH 7.0), the sample was loaded onto the column, and the column was washed with 20 volumes of the same buffer and inverted. For elution, a linear gradient toward a final concentration of 0.7 M NaCl in the same buffer over 20 column volumes was applied, and 1 mL fractions were collected.

Fractions were tested for 7β-hydroxysteroid dehydratase activity, and active fractions were subjected to native PAGE. Gels consisted of a gradient from 7% over 9–15% acrylamide (acrylamide/bisacrylamide 37.5:1) in Tris-HCl buffer (final concentration 325 mM, pH 8.8) with 0.2% ammonium peroxodisulfate and 0.02% *N*,*N*,*N*′,*N*′-tetramethylendiamine. 25 mM Tris with 192 mM glycine at pH 8.2 was used as running buffer and 100 mM Tris-HCl (pH 6.8) with 50% glycerol was used as loading buffer. Samples were loaded onto the gel two times and symmetrically, so that the gel could be cut into two identical halves after separation. One half was stained using Coomassie blue to identify protein bands and used as an orientation to cut the corresponding areas from the second, unstained half of the gel.

Protein bands were then incubated in 800 µL 50 mM MOPS buffer (pH 7.8) at room temperature and 1,200 rpm for 30 min. Supernatants were then used for finding bands with 7β-hydroxysteroid dehydratase activity. Protein bands from the stained gel corresponding to bands with activity were then analyzed by peptide mass fingerprinting.

### Purification of Hsh3 and Hsh2

For purification of Hsh3 using a his-tag, *hsh3* was cloned into the vector pET28(+)B using primer pair pur_for/rev for amplification and primer pair T7_for/rev for sequencing. pET28(+)B::*hsh3* was transferred into *E. coli* Tuner(BL3).

Two 250 mL cultures of each *E. coli* Tuner(BL3) pET28(+)B::*hsh3* (this study) and *E. coli* Tuner(BL3) pET28(+)B::*hsh2* (20) were inoculated in Erlenmeyer flasks from the respective pre-cultures at an initial OD_600_ of 0.02 and incubated at 37°C and 120 rpm for 2 h. 0.2 mM IPTG was added, and the cultures were further incubated at room temperature and 120 rpm overnight. Cells were harvested by centrifugation at 4°C and 8,000 *× g* for 8 min and kept on ice. Cells were washed and resuspended in 10 mL phosphate-buffered saline and lysed by ultrasonication (UP200S, Hielscher Ultrasonics, Teltow, Germany) with amplitude 60% and cycle 0.5 for 10 min on ice with 1 min breaks every 4 min. Cell debris was removed by centrifugation at 4°C and 20,000 × *g* for 60 min. Protein concentration in the cell-free extract was determined by BCA assay (Pierce, Thermo Fisher Scientific, Waltham, MA, USA).

Hsh3 and Hsh2 were purified using HisPur Ni-NTA spin columns (0.2 mL, Thermo Fisher Scientific, Waltham, MA, USA) according to the instructions. Columns were loaded with cell-free extracts, adding a total protein amount corresponding to two times the binding capacity of the column in two steps. Protein-containing fractions with concentrations > 10 mg mL^−1^ were further purified by gel filtration for imidazole removal. Gel filtration was performed using HiTrap desalting columns (Cytiva, Marlborough, MA, USA) according to the manual “operation with syringe” using 20 mM Tris buffer (pH 8) containing 150 mM NaCl. Fractions containing the protein were combined.

All purification steps were confirmed using SDS-PAGE.

### Enzyme assays

Enzyme assays were either conducted with purified Hsh3 and Hsh2 or with cell-free extracts. Cell-free extract was prepared as described (26). Strains were grown in 50 mL cultures in Erlenmeyer flasks and harvested in the exponential phase by centrifugation at 4°C and 8,000 *× g* for 10 min. The cells were washed and resuspended in 1.5 mL 50 mM MOPS buffer (pH 7.8), and lysed by ultrasonication (UP200S) with amplitude 60% and cycle 0.5 for 8 min on ice with 2 min after the first 4 min. Cell debris was removed by centrifugation at 4°C and 25,000 × *g* for 30 min.

Enzyme assays were performed in 50 mM MOPS buffer (pH 7.8) at 30°C. Enzyme assays with cell-free extracts contained 0.5 mM Δ^4^-3-keto-UDCA and 0.7 mg ml^−1^ total protein. Activity was determined by HPLC-MS measurement of samples taken at different time points. For all steps of biochemical purification, enzyme activity was tested using enzyme assays with Δ^4^-3-keto-UDCA (V). Substrate concentrations ranged from 0.15 mM in most assays to 0.036 mM in assays after ion exchange chromatography. In total, 0.1 mg mL^−1^ total protein or up to 40 µL of fractions after ion exchange chromatography was used for the assays. Enzyme activity in this case was monitored at 290 nm in a spectrophotometer during incubation at 30°C for 10 min (absorption coefficient of Δ^4,6^-3-keto compounds at 290 nm: 21.1 mol^−1^ cm^−1^ [66]). Activity of purified Hsh3 and Hsh2 was analyzed in either discontinuous or continuous assays, which were either monitored by HPLC-MS measurements of samples taken at different time points, or monitored spectrophotometrically, respectively. For discontinuous assays, 0.15 mM up to 1 mM substrate as well as 5 µg mL^−1^ up to 200 µg mL^−1^ protein were added. For continuous assays, 50 µM up to 1 mM substrate and 1 µg ml^−1^ up to 5 µg ml^−1^ protein were added, and absorption at 300 nm was measured against an assay solution without enzyme. Substrates for these assays were 7α-HADD (XI) and 7β-HADD, which were solved in ethanol. Activity in these assays was monitored photometrically at 300 nm (absorption coefficient of Δ^1,4,6^-3-keto compounds at 300 nm: 13.2 mol^−1^ cm^−1^ [20, 67]). Therefore, ethanol was added to all assays so that the final concentration was always 25%.

### Preparation of steroid compounds

UDCA was purchased from ChemPUR (Karlsruhe, Germany), CDCA from Carl Roth (Karlsruhe, Germany), and cholate from Sigma Aldrich (St. Louis, Mo, USA). Δ^4^-3-keto-UDCA (V) and Δ^4^-3-keto-CDCA (IV) were produced biotechnologically from UDCA and chenodeoxycholate as described previously (26). For this, 1 mM of the respective bile salt was transformed to the Δ^4^-3-keto derivative by strain Chol1 Δ*stdA1* Δ*kstD1* with additional 12 mM succinate until all substrate was transformed. Products were purified from culture supernatants by organic extraction with ethyl acetate after acidification. 7α-hydroxy ADD (7α-HADD, XI) and 7β-hydroxy ADD (7β-HADD) were produced biotechnologically from CDCA and UDCA, respectively. For this, 1 mM of the respective bile salt was transformed with strain Chol1 Δ*kshA* (31). Cultures were incubated until all substrate was transformed, and products were purified from culture supernatants by organic extraction with dichloromethane.

For purification of the steroid compound P2, cultures of strain Chol11 Δ*hsh3* were grown with 1 mM UDCA until only P2 was found in the supernatant. The compound was extracted by organic extraction with ethyl acetate after acidification with HCl. It was resolved in H_2_O_MQ_, and pH was adjusted with NaOH. For testing the stability of P2 under different conditions, the pH in aliquots was adjusted to <2 and >12 using HCl and NaOH, respectively. Further purification of P2 for NMR measurements was achieved by semi-preparative HPLC. It was then extracted from the eluate using organic extraction, resolved in methanol, and dried.

Concentrations of steroid solutions were determined photometrically using the respective absorption coefficients depending on the steroid nucleus configuration (Δ^4^-3-keto and Δ^1,4^-3-keto compounds: 14.7 mol^−1^ cm^−1^, Δ^4,6^-3-keto compounds: 21.1 mol^−1^ cm^−1^, Δ^1,4,6^-3-keto compounds: 13.2 mol^−1^ cm^−1^ (28, 66, 67).

### Analytical methods

Supernatants of steroid-containing samples were analyzed as described (26, 49) by HPLC-MS measurements after centrifugation for 5 min at >16,000 × *g* at room temperature.

HPLC-MS measurements were performed using a Dionex Ultimate 3,000 HPLC (ThermoFisher Scientific, Waltham, Massachusetts, United States) with an UV/visible light diode array detector and a coupled ion trap mass spectrometer (Amazon speed, Bruker; Bremen, Germany) with an electro-spray ion source (ESI). Separation was achieved over a reversed phase C18 column (Eurospher II 100–5, 150 × 3 mm, 5 µm particle size; Knauer, Berlin, Germany) at 25°C. 20 µL samples were injected. Either a method using ammonium-acetate buffer (10 mM, pH 6.7) and acetonitrile at a flow rate of 0.3 mL min^−1^ with a gradient from 10% to 48% acetonitrile (49), or a method using ammonium-acetate buffer (10 mM) with 1% formic acid and acetonitrile at a flow rate of 0.3 mL min^−1^ with a gradient from 10% to 90% acetonitrile (26) was used. For MS detection, the following settings were used: ultra-scan mode, scan range of 50–1,000 Da, dry gas flow 12 L min^−1^, dry gas temperature 200°C, nebulizer gas 22.5 psi, polarity alternating, end plate offset 500 V, and capillary voltage 4,000 V.

Steroid compounds were identified by mass, UV spectrum, and comparison with standards.

### Protein identification

Protein identification was performed with SDS-PAGE as well as native PAGE-separated protein. Gel slices were cut into smaller pieces (about 1 mm^2^) prior to washing, reduction, alkylation, and tryptic digestion (68). Separation of peptides was performed with a nano-LC system (Ultimate 3000 nanoRSLC, Thermo Fisher Scientific, Dreieich, Germany) operated in trap-column (3 µm beads, 75 µm inner diameter, 2 cm length; Thermo Fisher Scientific) mode. Separation was achieved with a 25 cm column (75 µm inner diameter, 2 µm beads; Thermo Scientific) applying a linear 90 min gradient from 2% vol/vol acetonitrile to 50% vol/vol acetonitrile with subsequent re-equilibration. The nanoLC eluent was continuously analyzed by an online coupled ion-trap mass spectrometer (amaZon speed ETD; Bruker Daltonik GmbH, Bremen, Germany) via an electrospray ion source (captive spray ion source; Bruker Daltonik GmbH) operated in positive mode. Per full scan MS, 20 tandem MS spectra of the most intense masses were acquired (precursor charge 2 + or more) with subsequent active precursor exclusion for 0.2 min. Protein identification was performed with the ProteinScape platform (version 3.1; Bruker Daltonik GmbH) on an in-house Mascot server (version 2.3; Matrix science, London, UK) against a genomic database of *Sphingobium* sp. strain Chol11 translated into amino acids, applying a target decoy strategy, including a mass tolerance of 0.3 Da for MS and 0.4 Da for MS/MS searches and applying a target decoy strategy (false discovery rate <1%) (69).

### NMR analysis

NMR analysis was performed using a Bruker NEO 500 MHz spectrometer equipped with a cryogenically cooled Prodigy HCN-TCI probe. A total of 5 mg of dry sample was dissolved in 280 µL of the deuterated methanol (Deutero GmbH, Kastellaun, Germany) and transferred to a 5 mm Shigemi NMR tube (Shigemi Co. Ltd, Tokyo, Japan) matched for methanol. All NMR spectra were acquired at 298 K and all chemical shifts were referenced relative to the tetramethylsilane signal. Structure elucidation was done using 1D ^1^H and ^13^C, as well as 2D ^1^H-^13^C HSQC, ^1^H-^13^C HMBC, 1,1-ADEQUATE, COSY, TOCSY, and NOESY experiments. Spectral windows for 1D experiments were 20 ppm for ^1^H and 20 ppm for ^13^C. For 2D experiments, the ^1^H spectral window was 12 ppm, and the ^13^C spectral window was 165 ppm for HSQC and 220 ppm for HMBC experiments, respectively. 1D spectra had 65536 data points, and 2D spectra had up to 4096 data points in the direct dimension and up to 1024 data points in the indirect dimension. NOESY mixing time was set to 500 ms. All NMR spectra were acquired, processed, and analyzed with Bruker TopSpin v4.1.3 software.

### Bioinformatic methods

Primers for unmarked gene deletion were designed using primerBLAST (70). Hsh3 homologs in other strains and in the database of the human genome project (54) were identified using Hsh3 and BLASTp (64, 71). Putative BaiI homologs in strain Chol11 were also identified using BLASTp. The sequence of Hsh3 was analyzed for domains and protein families using NCBI CDD and InterPro (72, 73). Similarities between Hsh3 and other proteins were calculated using global alignments (71, 74). Hsh3 and Hsh2 homologs in metagenomes of the MGnify database were identified using the MGnify website (75) and phmmer (76). The structure of Hsh3 was predicted using AlphaFold3 with the standard parameters (77) and visualized from these data using ChimeraX (78).

## SUPPLEMENTAL MATERIAL

Supplemental material aem.00185-25-s0001.pdf Tables S1 and S2, Text S1, and Figures S1 to S11.

ASM does not own the copyrights to Supplemental Material that may be linked to, or accessed through, an article. The authors have granted ASM a non-exclusive, world-wide license to publish the Supplemental Material files. Please contact the corresponding author directly for reuse.
