# Supplementary material for: 7β-Hydroxysteroid dehydratase Hsh3 eliminates the 7-hydroxy group of the bile salt ursodeoxycholate during degradation by Sphingobium sp. strain Chol11 and other Sphingomonadaceae
Source: Appl Environ Microbiol. 2025 May 9;91(6):e00185-25. doi: 10.1128/aem.00185-25 (PMC12175502; doi:10.1128/aem.00185-25)
Supplement: Supplemental material — Tables S1 and S2, Text S1, and Figures S1 to S11. [file aem.00185-25-s0001.pdf]

7β-hydroxysteroid dehydratase Hsh3 eliminates the 7-hydroxy group of the bile salt ursodeoxycholate during degradation by *Sphingobium* sp. strain Chol11 and other *Sphingomonadaceae*

Running title: 7β-hydroxysteroid dehydratase Hsh3

Phil Richtsmeier, Ruslan Nedielkov, Malte Haring, Onur Yücel, Lea Elsner, Rebekka Herdis Lülf, Lars Wöhlbrand, Ralf Rabus, Heiko Moeller, Bodo Philipp, Franziska Maria Mueller

Table S1: Structures, abbreviations, and molecular mass of all steroid compounds mentioned in this manuscript. In mass spectra $[X\text{-}H]^+$ is usually found as the main peak. In addition, $[2X\text{-}H]^+$ as well as adducts with ions from the buffer can be found. For bile salts and other compounds with carboxyl groups, the mass of the deproponated compound is given. Detailed information for new products P1 and P2 can be found in Fig S6 and Fig 6, respectively.

Number	Name	Abbreviation	Mass [Da]
I	Chenodeoxycholate	CDCA	391
II	Ursodeoxycholate	UDCA	391
III	Lithocholate	LCA	375
IV	Δ^4-3-Ketochenodeoxycholate	Δ^4-3-Keto-CDCA	387
V	Δ^4-3-Ketoursodeoxycholate	Δ^4-3-Keto-UDCA	387
VI	Δ^4-3-Ketolithocholate	Δ^4-3-Keto-CDCA	371
VII	3-Oxo-chol-4,6-diene-oate	OCDA	369
VIII	3-Oxo-chol-1,4,6-triene-oate	-	367
IX	$\Delta^{1,4}$-3-Ketolithocholate	$\Delta^{1,4}$-3-Keto-LCA	369
X	$\Delta^{1,4}$-3-Ketochenodeoxycholate	$\Delta^{1,4}$-3-Keto-CDCA	385
XI	7α-Hydroxy -androsta-1,4-diene-3,17-dione	7α-HADD	300
XII	3,7α-Dihydroxy-9,10-*seco*-androsta-1,3,5-triene-9,17-dione	3,7α-DHSATD	316
XIII	3´,5-Dihydroxy-3aα-*H*-4α(3´-propanoate)-7aβ-methylhexahydro-1-indanone	DH-HIP	253
XIX	3´-Oxo-5-hydroxy-3aα-*H*-4α(3´-propanoate-CoA)-7aβ-methylhexahydro-1-indanone	-	251+CoA
XX	5-Hydroxy-3aα-*H*-4α(3´-carboxyl-CoA)-7aβ-methylhexahydro-1-indanone	-	209+CoA
XXI	3-Hydroxy-9,10-*seco*-androsta-1,3,5(10),6-tetraene-9,17-dione	HSATD	298
XXII	7β-Hydroxy -androsta-1,4-diene-3,17-dione	7β-HADD	300
XXIII	7β-Hydroxy-3-oxo-pregna-1,4-diene-carboxylate	7β-Hydroxy-OPDC	257
XXIV	3,4,7β-Trihydroxy-9,10-*seco*-androsta-1,3,5-triene-9,17-dione	3,4,7β-THSATD	332

Text S1:

Bioinformatical prediction of other putative steroid dehydratases using Hsh2 (Yücel *et al.*, 2016) and the putative 7β-hydroxysteroid dehydratase BaiI from *Clostridium scindens* VPI12708 (Ridlon *et al.*, 2006) as query for BLASTp analyses resulted in the identification of seven candidate proteins (Table S2). Additionally, one protein belonging to the same NTF2-like superfamily as Hsh2 was found to be encoded in close vicinity to *hsh2*. Genes for all proteins mentioned in Table S2 were cloned into an *E. coli* expression strain. However, this did not result in the production of any enzyme with activity towards the substrate Δ^4-3-ketoursodeoxycholate (not shown).

Table S2: Bioinformatical prediction of 7β-hydroxysteroid dehydratase candidate genes in *Sphingobium* sp. strain Chol11. Most candidates: Homologs of predicted 7β-hydroxysteroid dehydratase BaiI from *Clostridium scindens* VPI12708 (UniProt-ID P32371). Candidate Nov2c408 was not found as a homolog of BaiI, but is from the same family as 7α-hydroxysteroid dehydratase Hsh2 (NTF-like superfamily) and encoded close to *hsh2.* (Nov2c400)

Protein	RefSeq-ID	Identity
**Nov1c358**	WP_097091110.1	26%
**Nov1c1702**	WP_097092250.1	33%
**Nov1c1795**	WP_097092893.1	30%
**Nov2c5**	WP_097093354.1	29%
**Nov2c89**	WP_097093425.1	42%
**Nov2c179**	WP_097093661.1	22%
**Nov2c373**	WP_176502263.1	28%
**Nov2c408**	WP_097092974.1	16%

References:

Yücel, O., Drees, S., Jagmann, N., Patschkowski, T., Philipp, B., 2016. An unexplored pathway for degradation of cholate requires a 7α-hydroxysteroid dehydratase and contributes to a broad metabolic repertoire for the utilization of bile salts in *Novosphingobium* sp. strain Chol11. Environ. Microbiol. 18, 5187–5203.

Ridlon, J.M., Kang, D.-J.J., Hylemon, P.B., 2006. Bile salt biotransformations by human intestinal bacteria. J. Lipid Res. 47, 241–259.

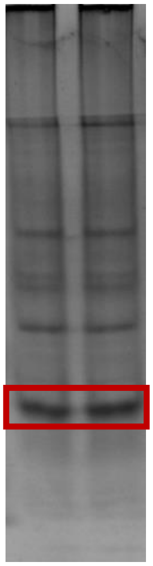

Figure S1: Native PAGE of protein fraction enriched with Hsh3 from *Sphingobium* sp. strain Chol11. Marked in red, bands with Hsh3 activity that were analyzes by protein mass fingerprinting.

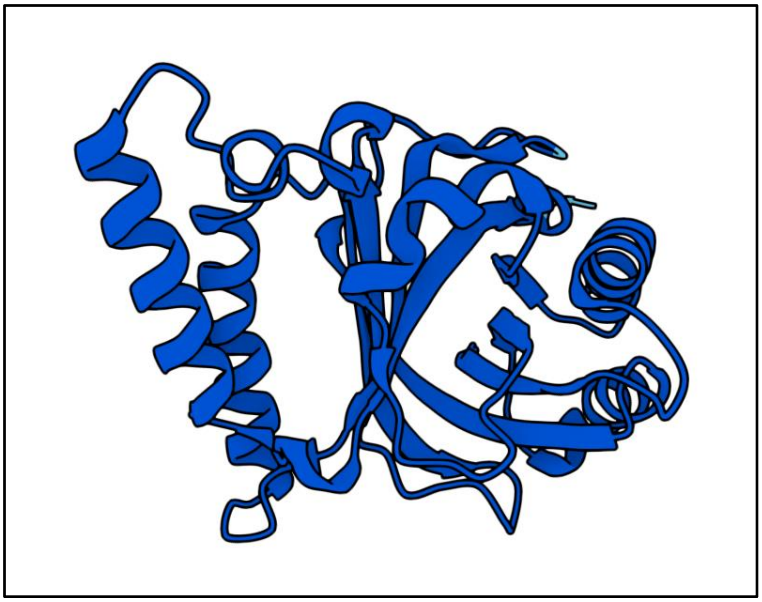

Figure S2: Hsh3 structure as predicted by Alphafold, coloring indicates local confidence as determined by predicted local distance difference test (plDDT). Dark blue (most of the structure), very high confidence (plDDT>90); cyan, confident (90>plDDT>70).

Strain	Chol1 Δ*stdA1* Δ*kstd1* pBBR1MCS-5::*hsh3*	Chol1 Δ*stdA1* Δ*kstd1* pBBR1MCS-5::*hsh3*	Chol1 pBBR1MCS-5::*hsh3*	Chol1 pBBR1MCS-5::*hsh3*	Chol1 pBBR1MCS-5::*hsh3* (several days old)
Color	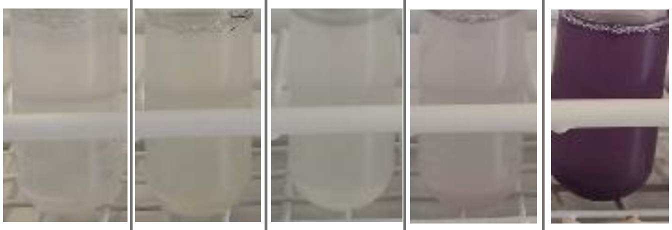				
Substrate	CDCA	UDCA	CDCA	UDCA	UDCA
Main product	Δ^4-3-keto-CDCA	OCDA	none	HSATD	HSATD

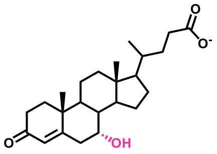

IV
Δ^4-3-Keto-CDCA

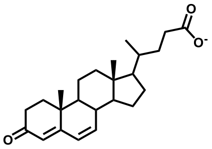

VII
OCDA
3-Oxo-chol-4,6-diene-oate

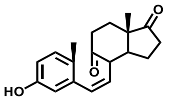

XXI HSATD
3-hydroxy-9,10-*seco*-androsta-1,3,5(10),6-tetraene-9,17-dione

Figure S3: Formation of $\Delta^{4,6}$-3-keto-steroid compounds and purple pigments in cultures of *P. stutzeri* Chol1 expressing *hsh3* from *Sphingoboium* sp. strain Chol11 and growing with UDCA. Purple color formation occurred after several days of incubation. Additionally shown are the main products found in the respective cultures.

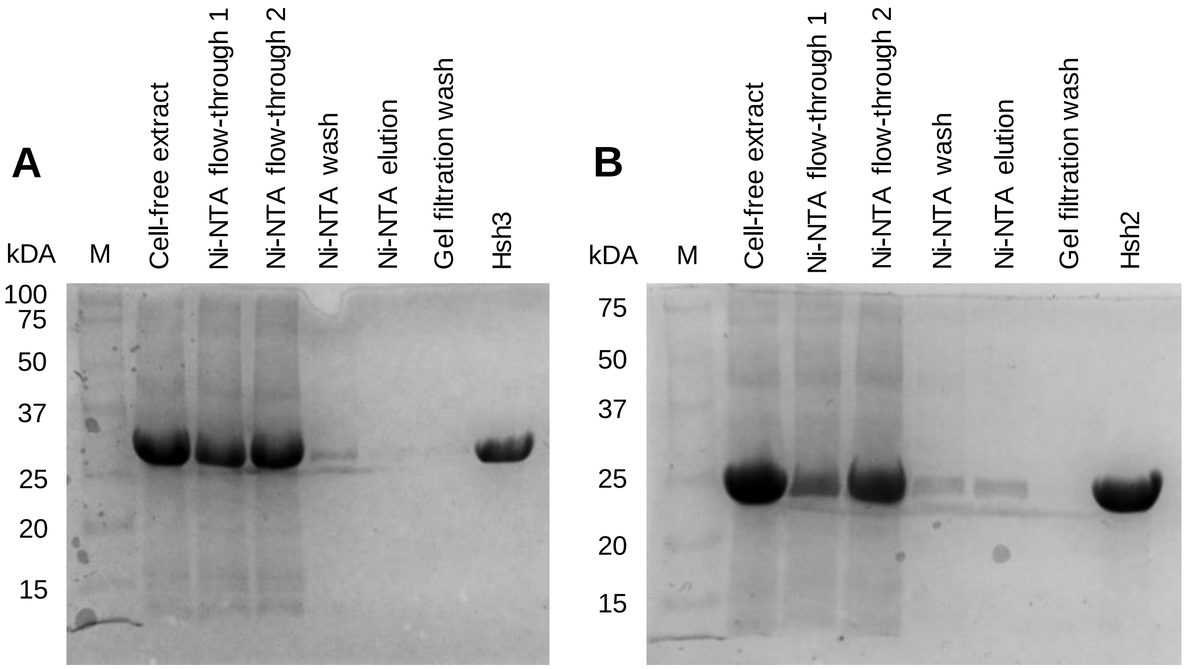

Figure S4: Purification of Hsh3 (A) and Hsh2 (B) from *Sphingobium* sp. strain Chol11. M, marker. Cell-free extract, cell-free extract of E. coli pET28B::*hsh3* or pET28B::*hsh2*,respectively. Ni-NTA flow-through, flow-through of Ni-NTA columns after adding cell-free extracts; Ni-NTA wash, flow-through of washing the Ni-NTA column; Ni-NTA elution, purified protein after elution from the Ni-NTA column; Gel filtration wash, wash step of gel filtration; Protein, purified protein after Ni-NTA purification and subsequent gelfiltration. Theoretical molecular weights of Hsh2 and Hsh3: 26.8 kDa and 20.3 kDA, respectively. Sample preparation: 1:10 dilution and incubation at 100 °C in denaturation buffer.

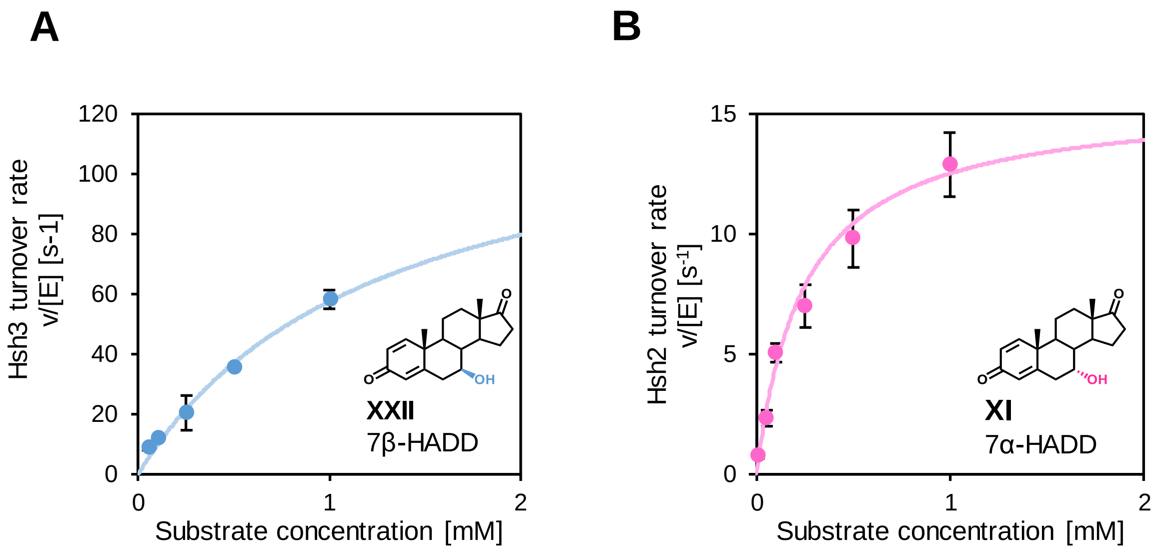

Figure S5: Turnover rate (enzyme activity v per enzyme concentration [E]) of purified Hsh3 (A) and Hsh2 (B) from *Sphingobium* sp. strain Chol11 at different substrate concentrations of 7β-HADD and 7α-HADD, respectively. Blue and red, measured data points; light blue and red, fit according to Michaelis-Menten kinetics. n=3, error bars indicate standard deviation.

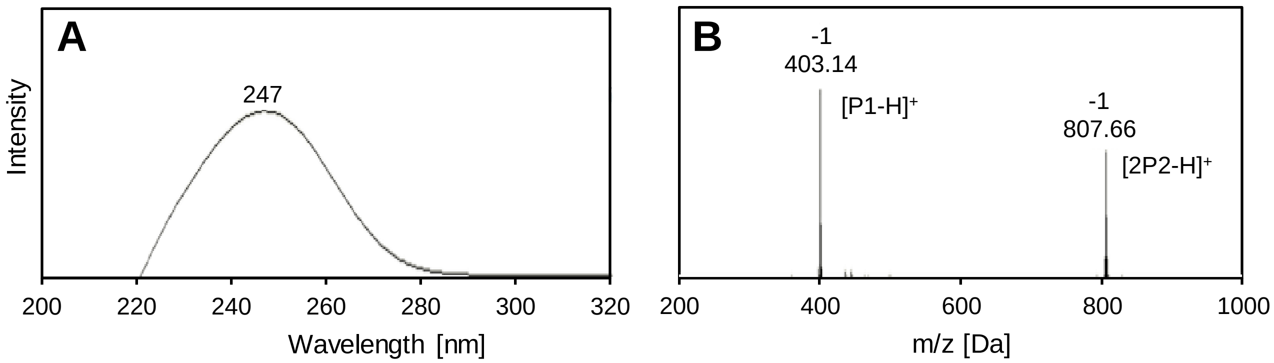

Figure S6: UV spectrum (A) and MS spectrum (B) of compound P1 accumulating transiently in the supernatant of *Sphingobium* sp. strain Chol11 Δ*hsh3* during growth with UDCA.

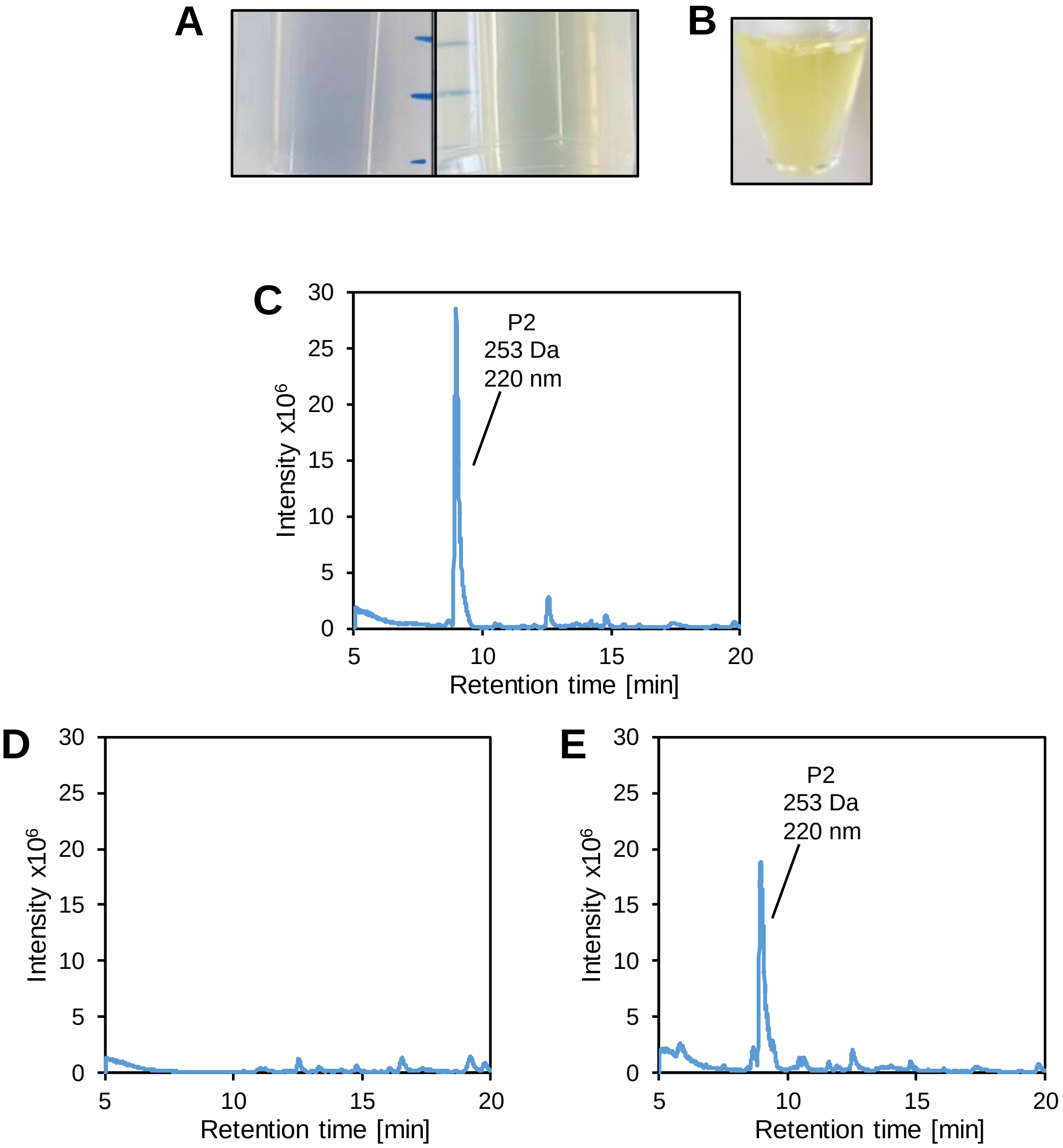

Figure S7: Characteristics of compound P2 formed by *Sphingobium* sp. strain Chol11 Δ*hsh3* from UDCA. (A) Supernatants of *Sphingobium* sp. strain Chol11 wt (left) and *Sphingobium* sp. strain Chol11 Δ*hsh3* (right). **(B)** Extracted P2 from *Sphingobium* sp. strain Chol11 Δ*hsh3* supernatant. **(C)** P2 in the culture supernatant at the end of the growth. **(D)** The same supernatant after addition of NaOH to a pH >12. **(E)** The same supernatant after addition of HCl to a pH <2. MS base peak chromatograms in negative mode.

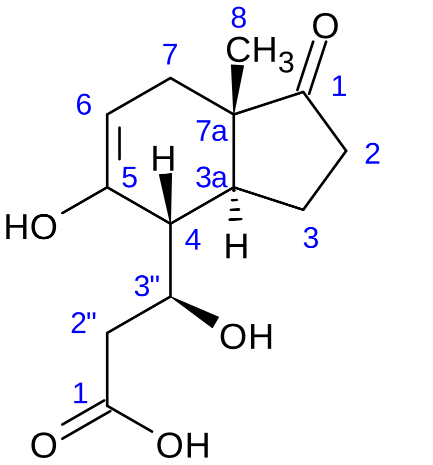

Molecular Formula: $C_{13}H_{18}O_5$

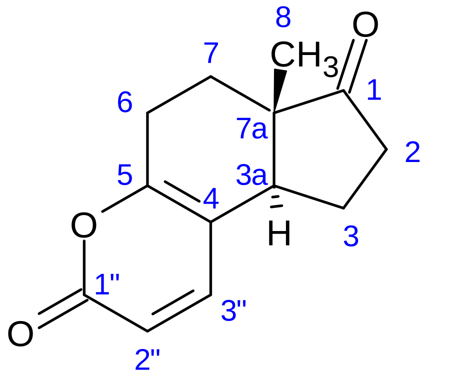

Molecular Formula: $C_{13}H_{14}O_3$

	**P2A**		**P2B**	
Atom no.	δ^{13}C (ppm)	δ^{1}H (ppm), $^{3}J_{HH}$ [a]	δ^{13}C (ppm)	δ^{1}H (ppm), $^{3}J_{HH}$
1	224.6	--- [b]	221.7	---
2	38.3	2.18, m<br>2.46, m	38.4	2.33, m<br>2.65, m
3	26.9	1.90, m<br>2.50, m	23.3	1.86, m<br>2.40, m
3a	48.3	1.90, m	46.0	2.90, dd (12.7/5.9 Hz)
4	45.6	2.31, m	118.9	---
5	136.2	---	143.4	---
6	106.9	5.12, dt (5.4/2.3 Hz)	26.7	2.81, m
7	33.4	2.02, m<br>2.10, m	29.8	1.73, m<br>1.98, m
7a	49.6	---	49.9	---
8	16.4	0.96, s	14.4	0.81, s
1“	172.4	---	166.8	---
2“	43.5	2.44, dd (17.1/10.9 Hz)<br>2.68, dd (17.1/5.3 Hz)	118.9	6.40, d (9.2 Hz)
3“	71.1	3.78, td (10.8/5.2 Hz)	143.4	7.47, d (9.2 Hz)

[a] m, multiplet; s, singlet; d, doublet; dd; doublet of doublet; dt, doublet of triplet; td, triplet of doublets.

[b] no proton at a given position.

Figure S8: NMR analysis of compounds P2A and P2B formed by *Sphingobium* sp. strain Chol11 Δ*hsh3* from UDCA. Performed on Bruker NEO 500 MHz spectrometer equipped with a cryogenically cooled Prodigy HCN-TCI probe at 298 K using 1D ^{1}H and ^{13}C, as well as 2D ^{1}H-^{13}C HSQC, ^{1}H-^{13}C HMBC, 1,1-ADEQUATE, COSY, TOCSY and NOESY experiments and P2A and P2B solved in deuterated methanol.

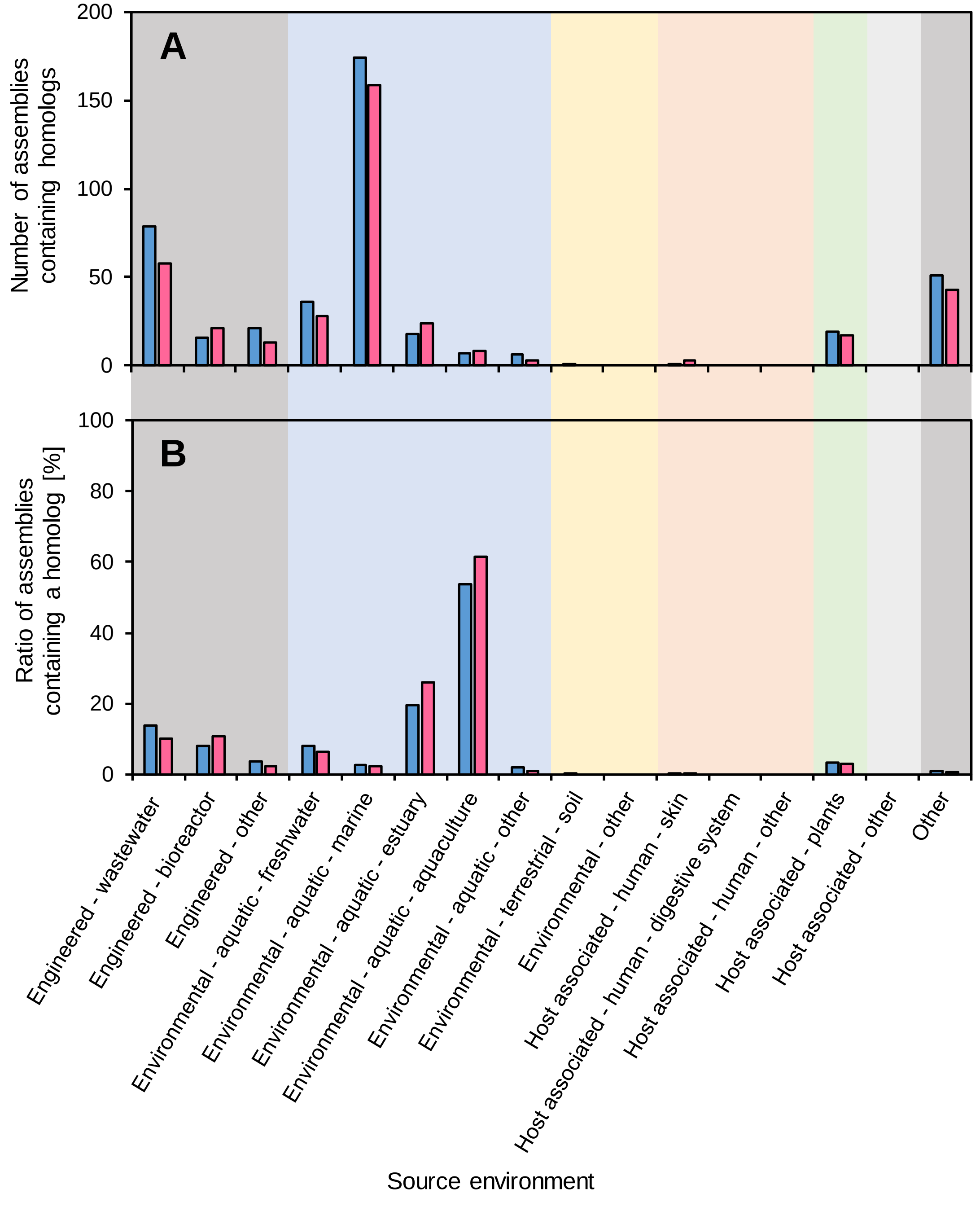

Figure S9: Homologs of Hsh3 (blue) and Hsh2 (red) from *Sphingobium* sp. strain Chol11 in metagenomes from different environments. Number (A) and percentage (B) of assemblies from the MGnify metagenome database that contain homologs (e-value ≤ 10^{-25}). Bars indicate individual data points.

Strain	Organism	Identity	E-value	Final OD_{600}	Products
E2U	Comamonas thiooxydans	99.92	0	0.7	No products; intermediate 7β-hydroxy-OPDC
E3U	Sphingobium japonicum	98.39	0	0.7	No products; intermediate OCDA
R1U	Comamonas testosteroni	100	0	0.7	No products; intermediate 7β-hydroxy-OPDC
R2U	Comamonas testosteroni	100	0	0.7	No products; intermediate 7β-hydroxy-OPDC
R3U	Comamonas testosteroni	100	0	0.7	No products; intermediate 7β-hydroxy-OPDC
P1U	Comamonas testosteroni	99.92	0	0.8	No products; intermediate 7β-hydroxy-OPDC
P2U	Comamonas testosteroni	100	0	0.7	No products; intermediate 7β-hydroxy-OPDC
P3U	Comamonas testosteroni	100	0	0.7	No products; intermediate 7β-hydroxy-OPDC
W11U	Novosphingobium aquaticum	98.51	0	0.55	No products; intermediate 3-keto-$\Delta^{4,6}$-compound, unknown mass
W22U	Comamonas testosteroni	99.93	0	0.2	7β-hydroxy-OPDC, 3,7β-DHSATD, 3,4,7β-THSATD
W24U	Comamonas testosteroni	100	0	0.7	No products; intermediate 7β-hydroxy-OPDC
W32U	Pseudomonas putida	100	0	0.6	No products; intermediate 7β-hydroxy-OPDC and other 3-keto-$\Delta^{1,4}$-compound
W52U	Pseudomonas entomophila	99.93	0	0.55	No products; intermediate 7β-hydroxy-OPDC and other 3-keto-$\Delta^{1,4}$-compound
W61U	Novosphingobium subterraneum	98.07	0	0.55	No products; intermediate 3-keto-$\Delta^{4,6}$-compound, unknown mass

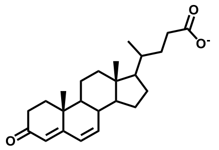

VII
OCDA
(3-Oxo-chol-4,6-diene-oate)

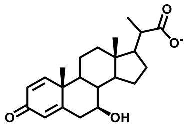

XXIII
7β-hydroxy-OPDC
(7β-hydroxy-3-oxo-pregna-1,4-diene-carboxylate)

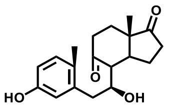

XII
3,7β-DHSATD
(3,7β-Dihydroxy-9,10-*seco*-androsta-1,3,5-triene-9,17-dione)

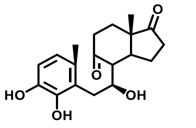

XXIV
3,4,7β-THSATD
(3,4,7β-Trihydroxy-9,10-*seco*-androsta-1,3,5-triene-9,17-dione)

Figure S10: Environmental isolates enriched using UDCA as only carbon source. Organism, closest relative according to BLASTn search of the 16s rRNA genes; identity, similarity of the 16s rRNA genes sequence of isolate and strain found in the database. Final OD_{600}, intermediates, and products after growth with 1 mM UDCA. No products indicates no detectable products found in the supernatant at the end of growth, intermediate indicates compound accumulated transiently during growth that are degraded again.

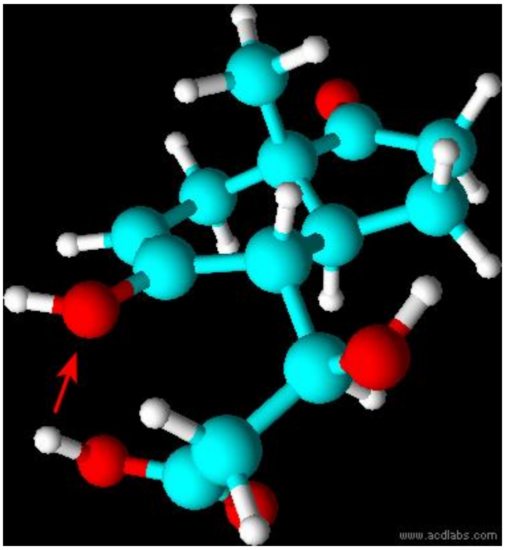

Figure S11: Potential formation of an intramolecular hydrogen bond in compound P2A formed by *Sphingobium* sp. strain Chol11 Δ*hsh3* from UDCA that can stabilize the enol structure. Red arrow, position of the hydrogen bond.
